# Evolution of mouse circadian enhancers from transposable elements

**DOI:** 10.1186/s13059-021-02409-9

**Published:** 2021-06-29

**Authors:** Julius Judd, Hayley Sanderson, Cédric Feschotte

**Affiliations:** 1 Department of Molecular Biology and Genetics, Cornell University, Ithaca, NY 14853 USA; 2 Department of Human Genetics, University of Utah School of Medicine, Salt Lake City, UT 84112 USA

**Keywords:** Transposable elements, Enhancers, Transcription, Gene regulation, Regulatory evolution, Circadian rhythms

## Abstract

**Background:**

Transposable elements are increasingly recognized as a source of *cis*-regulatory variation. Previous studies have revealed that transposons are often bound by transcription factors and some have been co-opted into functional enhancers regulating host gene expression. However, the process by which transposons mature into complex regulatory elements, like enhancers, remains poorly understood. To investigate this process, we examined the contribution of transposons to the *cis*-regulatory network controlling circadian gene expression in the mouse liver, a well-characterized network serving an important physiological function.

**Results:**

ChIP-seq analyses reveal that transposons and other repeats contribute ~ 14% of the binding sites for core circadian regulators (CRs) including BMAL1, CLOCK, PER1/2, and CRY1/2, in the mouse liver. RSINE1, an abundant murine-specific SINE, is the only transposon family enriched for CR binding sites across all datasets. Sequence analyses and reporter assays reveal that the circadian regulatory activity of RSINE1 stems from the presence of imperfect CR binding motifs in the ancestral RSINE1 sequence. These motifs matured into canonical motifs through point mutations after transposition. Furthermore, maturation occurred preferentially within elements inserted in the proximity of ancestral CR binding sites. RSINE1 also acquired motifs that recruit nuclear receptors known to cooperate with CRs to regulate circadian gene expression specifically in the liver.

**Conclusions:**

Our results suggest that the birth of enhancers from transposons is predicated both by the sequence of the transposon and by the *cis*-regulatory landscape surrounding their genomic integration site.

**Supplementary Information:**

The online version contains supplementary material available at 10.1186/s13059-021-02409-9.

## Background

Change in gene regulation is an important mechanism underlying the emergence of new biological traits [1–5]. There is a substantial body of empirical studies illustrating how the addition, modification, or disappearance of *cis*-regulatory elements, such as enhancers, has driven the emergence of profound phenotypic changes throughout evolution [6–8]. Thus, there has been an intensifying effort over the past decade to better understand mechanisms underlying the evolution of enhancers and other *cis*-regulatory elements [4, 9–12].

In the broadest definition, enhancers are short (100 bp–1 kb) DNA sequences that modulate transcription of target genes regardless of genomic orientation or distance, and are often bound by transcription factors (TFs) [13, 14]. Recent advances in functional genomics enabled nearly unbiased mapping of enhancers and their associated TF binding sites (TFBSs) on a genome-wide scale and facilitated systematic studies of enhancer evolution across and within species [11, 15–17]. Seminal comparative studies in mammals revealed a low level of conservation in the genomic location of enhancers relative to genes and their promoters [18–27]. For instance, Villar and colleagues found that nearly half of 20,000–25,000 active liver enhancers mapped in each of 20 mammalian species are lineage- or even species-specific, while almost all promoters active in the liver are conserved across most or all the species examined [28]. However, recent analyses demonstrated that deeply conserved enhancers often coordinate robust and essential gene expression programs while less conserved enhancers contribute plasticity and redundancy to gene regulatory networks [29–31]. While these studies point to the rapid turnover of enhancers during mammalian evolution, the mechanisms underlying the birth and death of enhancers are only beginning to be understood [10, 11, 26, 32–35].

Transposable elements (TEs) represent an important source of new *cis*-regulatory elements, including enhancers. TEs account for a substantial amount of nuclear DNA and genetic variation in virtually all metazoans [36]. For example, between one and two-thirds of all mammalian genomes thus far examined are recognizable as being derived from TE sequences [36–39]. These elements inserted at various times during mammalian evolution, ranging from highly decayed copies integrated > 100 million years ago to recently integrated copies that may be species-specific or still polymorphic in the population [37–42]. Several studies have systematically examined the contribution of TEs to TF binding and the birth of *cis*-regulatory elements, and some general principles have emerged [43–47]. First, TEs contribute a substantial but widely variable fraction (~ 2–40%) of the TFBSs mapped for a given TF throughout the genome [48–52]. Second, TFBSs and *cis*-regulatory elements derived from TEs tend to be evolutionarily recent and are restricted to specific species or lineages [34, 50, 53]. For example, ∼20% of OCT4 and NANOG binding sites were derived from lineage-specific TEs in humans and mice [20]. This may be explained by the fact that the majority of TEs in any mammalian genome are themselves lineage-specific: for example, 85% of mouse TEs are not shared with the human [40] and 35% are not even shared with the rat [54]. Third, not all TEs contribute equally: for any given TF, there is generally one or a few TE families that account for a disproportionate fraction of binding sites relative to their frequency in the genome [20, 44, 46, 48, 50, 51].

Multiple studies have now confirmed that different TE classes and families contribute TFBSs for different TFs in different mammalian species and that these TE-derived TFBSs occasionally undergo exaptation to give rise to new host regulatory elements (reviewed in [44, 47]). However, the mechanisms by which complex enhancers emerge from TEs remain poorly understood. For instance, it is unclear why specific TE families or copies are bound by a particular TF while closely related elements in the same genome are not [45, 51]. The path by which individual TE copies are co-opted for regulatory purposes has been scarcely characterized [55], and the relative contributions of combinatorial sequence motifs pre-existing within TEs or in the vicinity of their insertion sites have not been examined in detail. To address these and other poorly understood aspects of TE co-option in regulatory evolution, we chose to examine their contribution to the *cis*-regulatory network underlying circadian gene expression. The machinery responsible for the transcriptional control of circadian gene expression is deeply conserved and has been extensively characterized in the mouse liver, which provides a solid experimental framework against which the impact of TEs can be queried. The circadian clock also presents the relatively unique advantage of providing a particularly robust system to examine the binding of TEs by regulatory proteins, as circadian rhythms are maintained by a series of interconnecting feedback loops of paralogous TFs [56–58].

The primary feedback loop consists of six circadian regulators (CRs), of which two are transcriptional activators (BMAL1 and CLOCK) and four are transcriptional repressors (PER1, PER2, CRY1, and CRY2). During the day, BMAL1 and CLOCK form a heterodimer, which binds to a tandem pair of E-box motifs in distal and promoter regions of clock-controlled genes [59]. Among the direct targets of the BMAL1:CLOCK complex are the repressors PER1/2 and CRY1/2. Following translation, PER and CRY enter the nucleus and inhibit BMAL1:CLOCK mediated transcription, thereby decreasing their own transcription and generating a feedback loop essential to the maintenance of the clock period [60, 61]. This model has recently been revised to reflect reports that BMAL1 acts as a pioneer factor and promotes rhythmic nucleosome removal and that transcription promoted by CLOCK:BMAL1 is not homogeneously oscillatory [62]. It is proposed that CLOCK:BMAL1 binding rhythmically maintains a chromatin landscape which facilitates binding and transcriptional activation by other ubiquitous or tissue-specific transcription factors, including members of the nuclear receptor (NR) family [63]. Interactions between CRs and liver-specific NRs are thought to underlie liver-specific circadian regulation of metabolic processes such as glucose, cholesterol, and lipid metabolism [64–66]. The vast amount of data and knowledge available for circadian regulation in the mouse liver provides a solid paradigm to dissect the mechanisms underlying the contribution of particular TEs to this *cis*-regulatory network.

## Results

### Repetitive elements contribute CR TFBSs with weaker enhancer chromatin signatures than non-repetitive CR TFBSs

To investigate the contribution of DNA repeats to *cis*-regulatory elements entwined in the mammalian circadian gene regulatory network, we turned to a seminal collection of ChIP-seq experiments that reported the genome-wide binding profiles of the six core CRs in the mouse liver [67]. To utilize the most recent mouse genome annotation, we aligned raw sequencing reads (Table 1) to the mm10 genome assembly and used uniquely mapped reads to call peaks. Of the peaks identified with this approach, ~ 60–92% overlapped with peaks originally reported [67] (Additional file 1: Fig. S1A). Cognate sets of CLOCK and BMAL1 peaks overlapped by ~ 85% (Additional file 1: Fig. S1A). The fraction of peaks that did not overlap between the two sets (based on their distance from each other) were largely low confidence peaks (Additional file 1: Fig. S1B). The fold change over background of the re-called peaks correlated with the summit height of the original peaks (Additional file 1: Fig. S1C). Overall, the total number of peaks called for each CR was in good agreement with those reported in the published analysis [67] (Additional file 1: Fig. S1D). Together, these findings validate this set of CR binding sites in the mouse liver.

**Table 1:** Sources of data used in this study

Data	SRA	Citation
CLOCK, BMAL1, CRY1, CRY2, PER1, PER2 ChIP-seq	SRP014752	[67]
DNase-seq, H3K27Ac ChIP-seq, Pol II ChIP-seq	SRP045509	[68]
GRO-seq, RORα ChIP-seq	SRP044381	[69]
RXR, LXR, PPARα ChIP-seq	SRP010657	[70]
REV-ERBα, REV-ERBβ ChIP-seq	SRP009472	[71]
High confidence CLOCK:BMAL1 binding sites	N/A	[63]
Tissue-specific BMAL1 binding sites	SRP132869	[72]
Hepa 1-6 RNA-seq	SRP059935	[73]

**Fig. 1:**
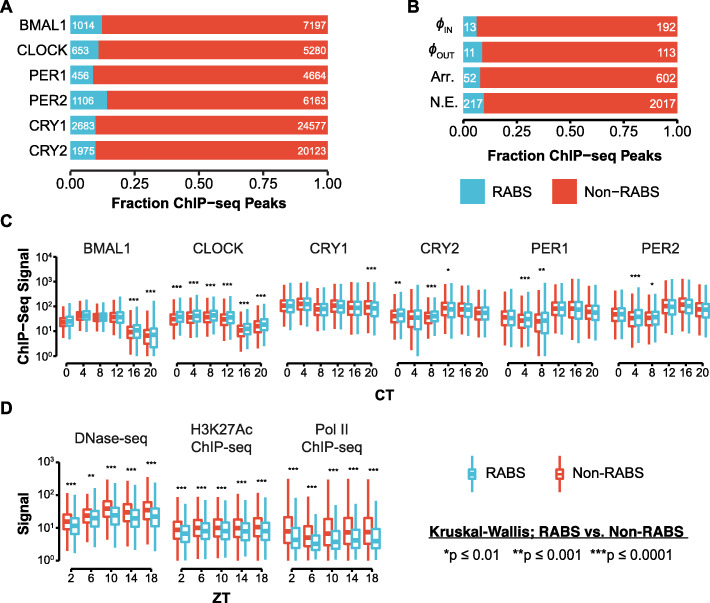
Repeat-derived CR binding sites display similar CR binding but less hallmarks of enhancer activity. **A** Fraction of ChIP-seq peaks for each CR for which > 50% of the peak did (RABS) or did not (Non-RABS) map within repetitive elements. **B** CLOCK:BMAL1 ChIP-seq peaks sorted by the nascent transcriptional output of the closest gene (rhythmic in-phase or out-of-phase; Arr, *Arr*hythmic; NE, *N*ot *E*xpressed), classified as RABS or Non-RABS as in **A**. **C** Distribution of ChIP-seq signal under RABS (n = 1014) and Non-RABS (n = 7197) BMAL1 ChIP-seq peaks across circadian time (CT). Significance testing compares RABS to Non-RABS at each timepoint. See Additional file 1: Fig. S2A for heatmaps of the ChIP-seq data that underlies these boxplots at the same sets of regions. Each boxplot represents the distribution of the total ChIP-seq signal under the respective set of peaks in the indicated dataset (sets of peaks are the same across timepoints and datasets). **D** Distribution of DNase-seq, H3K27Ac ChIP-seq, and Pol II ChIP-seq signal in RABS (n = 1014) and Non-RABS (n = 7197) BMAL1 ChIP-seq peaks across Zeitgeber time (ZT). Significance testing compares RABS to Non-RABS at each timepoint. See Additional file 1: Fig. S2B–D for heatmaps of the ChIP-seq data that underlies these boxplots at the same sets of regions. Each boxplot represents the distribution of the total ChIP-seq signal under the respective set of peaks in the indicated dataset (sets of peaks are the same across timepoints and datasets). Boxplots in **C** and **D** show the median (center line), the 1st and 3rd quartiles (hinges), and 1.5 × IQR (whiskers). Significance values are from the Kruskal-Wallis test with Bonferroni-corrected Dunn post hoc comparison

We then categorized CR binding sites as *R*epeat-*A*ssociated *B*inding Sites (RABS) [52] if the ChIP-seq peak coordinates showed positional overlap of > 50% with a repetitive element annotated by RepeatMasker, which was accessed from the UCSC genome browser and filtered for low complexity and simple repeats [74–76]. This analysis revealed that between 8.3 and 14.3% of each CR peak set mapped within TEs or other repetitive elements (Fig. 1A). To corroborate these results, we turned to a published set of stringent CLOCK:BMAL1 binding sites that was derived from the same ChIP-seq data but required occupancy by both CLOCK and BMAL1 and was categorized as rhythmic, arrhythmic, or not expressed based on the circadian nascent transcriptional output of the nearest gene [63]. Rhythmic peaks were further classified as transcriptionally in or out of phase respective to CLOCK:BMAL1 binding. Interestingly, when we intersected these peaks with the coordinates of repetitive elements as described above, we observed slightly lower percentages (6.3–9.7%) of RABS (Fig. 1B), which led us to speculate that TE-derived CR binding sites might be less likely to have biological relevance than non-repeat-derived CR binding sites.

To investigate the biochemical activity of RABS, we examined temporal genome-wide binding profiles of each CR. As a control, we selected a random set of repeats matching the familial composition of the RABS set. The binding pattern of CRs at RABS recapitulates that observed at Non-RABS: CLOCK and BMAL1 binding increases from CT0–CT8 and then tapers off, PER1/2 and CRY2 binding is initially low and increases from CT12–CT20, and CRY1 binding is maximally bound at CT0–CT4 but also increases at CT12 and CT20 (Fig. 1C; Additional file 1: Fig. S2A). When we quantified ChIP-seq signal within peaks, the only statistically significant difference consistent for all time points was that median CLOCK signal was increased in RABS relative to Non-RABS (Fig. 1C; compare red boxplot to blue boxplot at each timepoint). However, the magnitude of this increase is subtle and led us to conclude that, overall, the oscillatory profile of CR binding at RABS and Non-RABS is essentially indistinguishable.

The preferred binding motif of the CLOCK:BMAL1 dimer is a pair of E-Box motifs (CACGTG) separated by a variable 6-7 nucleotide spacer [59]. We predicted that both RABS and Non-RABS would be enriched in E-Box motifs, and found that there was indeed a strong pattern of E-Box enrichment at the center of both RABS and Non-RABS peaks, but no such enrichment at the center of our set of randomly selected repeats (Additional file 1: Fig. S3A). The average distance from RABS peaks and repeats to the closest E-Box was similar to that observed from Non-RABS peaks, and both sets are much closer than expected by chance (Additional file 1: Fig. S3B). Of E-Box motifs associated with RABS, 82.5% fell within the boundaries of the associated repeat, indicating that the repeats themselves are contributing to the binding site and are directly bound by CRs.

Because RABS are bound by CRs in a similar manner to Non-RABS, we would expect that they would have similar chromatin state and regulatory potential. To interrogate chromatin state and regulatory activity or CR binding sites, we used published H3K27Ac ChIP-seq, DNase-seq, and Pol II ChIP-seq experiments conducted in the mouse liver in Zeitgeber time [68], which is distinct from circadian time in that animals are harvested over a light-dark cycle rather than constant dark conditions. We accessed raw sequencing reads (Table 1) and processed them with the software pipeline used for CR ChIP-seq data with minor adjustments (see the “Methods” section). Intriguingly, while the oscillatory pattern of DNase sensitivity, H3K27 acetylation, and Pol II occupancy is maintained between RABS and Non-RABS, the median observed signal intensity is significantly lower at RABS than at Non-RABS (Fig. 1D). We confirmed this observation by visualizing individual loci as heatmaps (Additional file 1: Fig. S2B–D). The magnitude of these differences between RABS and Non-RABS is substantially greater than those observed in CR binding (Fig. 1C, D; compare red boxplots to blue boxplots at each timepoint). This could be a result of differences in the unique mappability of repetitive regions between experiments, but the DNase-seq, H3K27Ac ChIP-seq, and Pol II ChIP-seq libraries were sequenced with longer read length than the CR ChIP-seq libraries, so the unique mappability of these libraries should be better, not worse (Additional file 1: Fig. S2E).

We then used the Genomic Regions Enrichment of Annotations Tools (GREAT) [77] to examine whether the genes proximal to E-Box motifs within RABS and Non-RABS ChIP-seq peaks (two nearest genes within 1 Mb) are enriched for particular mouse phenotypes. GREAT determines enrichment by comparing the gene associations of input sites with those of a user-defined background. When we used the entire mouse genome as background, RABS and Non-RABS displayed significant enrichment for similar annotations corresponding to fairly broad ontologies, which are largely related to liver function or circadian phase as previously reported [67] (Additional file 1: Fig. S4A-B). We reasoned that these enrichments may be driven by the general open chromatin landscape of liver cells rather than by biological processes more specifically under circadian control in the liver. Indeed, when we repeated the GREAT analysis using all open chromatin regions—defined by DNase accessibility at any time point—as background, a different trend emerged. While Non-RABS were again enriched in phenotypes likely to be related to general liver function and circadian phase (Additional file 1: Fig. S4C), RABS were enriched in categories related to pigmentation phenotypes, including abnormal digit pigmentation, non-pigmented tail tip, and variable body spotting (Additional file 1: Fig. S4D). Collectively, these analyses indicate that RABS and Non-RABS share similar CR occupancy and motif composition, but RABS exhibit weaker signals of regulatory activity as a whole and are tied to genes whose expression patterns could be related to species-specific phenotypic variation.

### RABS are enriched for RSINE1 elements

**Fig. 2:**
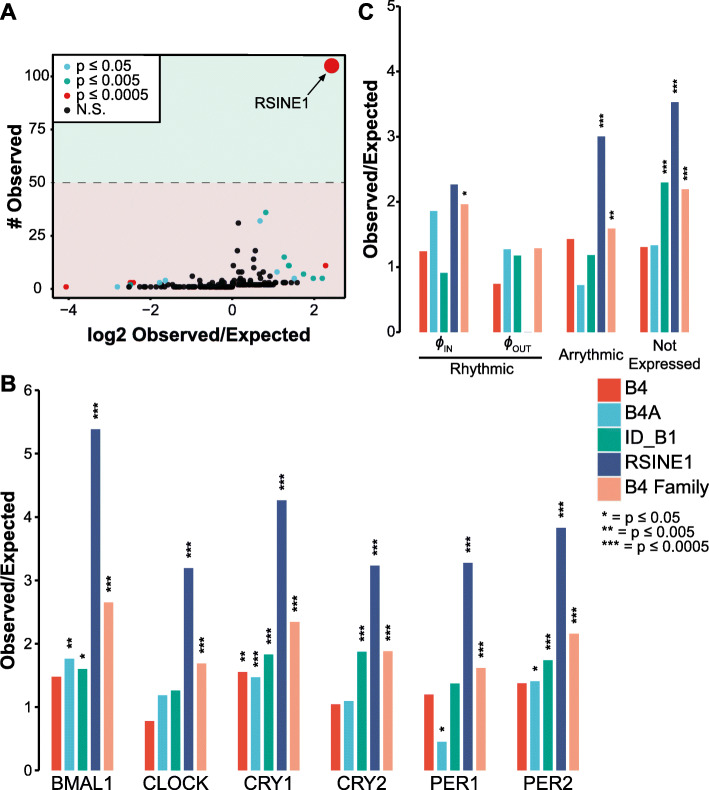
RSINE1 elements are overrepresented in RABS. **A** All TEs detected in BMAL1 ChIP-seq peaks, plotted by the number of elements observed compared to the ratio of observed to expected occurrences for that particular TE. Expected values were calculated by bootstrapping 1000 times (see the “Methods” section). The dashed line denotes our cutoff of > 50 elements observed imposed to filter false positives. **B** Ratio of observed to expected occurrences, as in **A**, for individual members of the B4 family and for the entire B4 family, in sets of RABS associated with each CR. **C** As in **B**, but enrichment of TEs in high confidence CLOCK:BMAL1 binding sites categorized by their nascent transcriptional output. All p-values were obtained using a two-sided binomial test

To determine if particular TEs were contributing CR RABS more often than expected from their frequency in the genome, we tested for enrichment of individual mouse TE families for their intersection with ChIP-seq peaks using a previously described pipeline [78]. We found that several TE families were significantly enriched in each CR dataset, but after imposing a cutoff of > 50 observed bound copies per family, only a single family, RSINE1, stood out across 5/6 ChIP-seq datasets (Fig. 2A). While RSINE1 did not meet our threshold of 50 elements bound in the PER1 ChIP-seq data with 43 unique elements bound, enrichment of RSINE1 was still statistically significant. For each set of CR binding sites, there were between 43 and 242 RSINE1-derived RABS, representing 3.2- to 5.4-fold enrichment over expectation (Fig. 2B). Importantly, other related B4 SINE families including B4, B4A, and ID_B1 were only significantly enriched for binding of 1–3 of the CRs and no consistent trend was apparent. In total, 328 unique RSINE1 elements out of 115,806 total RSINE1 elements in the genome were bound by at least one CR. Application of this method to CLOCK:BMAL1 peaks sorted by the nascent transcriptional output of the nearest gene [63] revealed similar levels of RSINE1 enrichment among “arrhythmic” and “not expressed” classes of binding sites, but no significant increase in RSINE1 elements in “rhythmic in-phase” transcriptionally cycling binding sites (Fig. 2C). No RSINE1 elements were associated with “rhythmic out-of-phase” transcriptionally associated binding sites. This corroborates our previous speculation that TE-derived CR binding sites are less likely to have biological relevance than non-RABS CR binding sites.

**Fig. 3:**
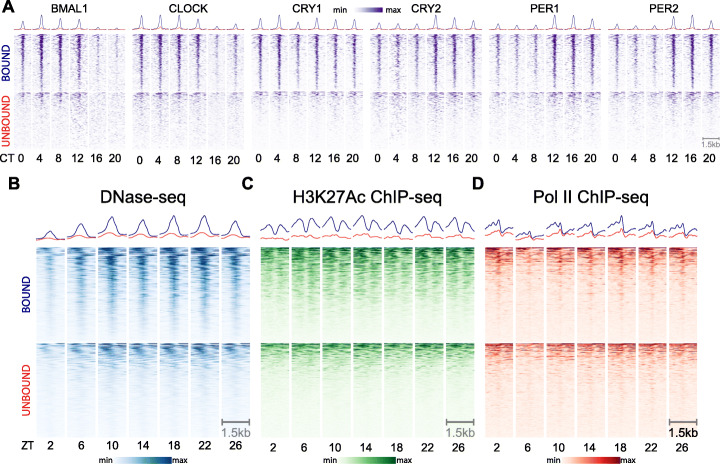
Differential CR occupancy, chromatin landscape, and Pol II occupancy of bound and unbound RSINE1 elements. **A** ChIP-seq signal for each CR across circadian time (CT), centered at CR associated RSINE1 elements (n = 328, top) and a randomly selected set of 328 RSINE1 elements which fall within open chromatin regions as defined by DNase-seq but are not associated with CR binding (n = 328, bottom). Heatmaps are centered at the middle of each element in positive orientation, extend 750 bp in each direction, and are sorted by mean signal intensity across all rows for a given CR. **B** DNase-seq signal over Zeitgeber time (ZT). Regions, sorting, and centering as in **A**. **C** H3K27Ac ChIP-seq signal over Zeitgeber time (ZT). Regions, sorting, and centering as in **A**. **D** Pol II ChIP-seq signal over Zeitgeber time (ZT). Regions, sorting, and centering as in **A**. In all panels, color scaling is relative to min-max signal within each block of heatmaps. Trace above heatmaps is the average signal in each class

To investigate why some RSINE1 elements are bound by CRs while others are not despite their identical sequence upon insertion, we first compared temporal CR binding patterns, chromatin landscape, and Pol II occupancy between RSINE1 elements predicted to be bound by CRs and those apparently unbound but still residing in open chromatin (OC; unbound). We reasoned that RSINE1 elements in open chromatin regions might be more informative for comparison than random elements, though by doing so we are more likely to introduce false negatives in our unbound set. We found that there were strong oscillatory patterns of CR binding enriched at these bound RSINE1s, and while the pattern of enrichment on the unbound fraction was also oscillatory, it was weaker and more diffuse (Fig. 3A). We verified that this is only true of CR-unbound OC RSINE1s by inspecting heatmaps of unbound RSINE1s that do not reside in open chromatin and observed no detectable oscillatory CR ChIP-seq signal on these elements (Additional file 1: Fig. S5). While this was somewhat surprising, we reasoned it could occur if these unbound OC RSINE1s were bound by other TFs themselves under circadian regulation or resided in genomic locale broadly regulated by CRs.

Furthermore, the vast majority of bound RSINE1s displayed patterns of DNase hypersensitivity (Fig. 3B), H3K27 acetylation (Fig. 3C), and Pol II occupancy (Fig. 3D) characteristic of active circadian enhancers. These patterns were rarely apparent, diffuse, and off-center relative to RSINE1 when examining unbound OC RSINE1s (Fig. 3B–D). While we expected that these OC RSINE1s would show DNase hypersensitivity—we selected this set precisely for this property—the oscillatory nature of the accessibility was surprising. We speculate this is driven by bound RSINE1s which did not meet the stringent 50% overlap criterion used in this analysis, and as such, our set of bound RSINE1s is likely an underestimate. Indeed, when we intersected our randomly selected set of unbound OC RSINE1 elements with BMAL1 ChIP-seq peaks, we found that 20/328 (6%) of these elements would have been classified as RABS if the overlap criterion was 25% instead of 50%. Taken together, these results indicate that RSINE1 elements contribute a disproportionate fraction of CR TFBSs in the mouse liver and that CR-bound RSINE1 elements display hallmarks of active regulatory elements.

### CR binding of RSINE1s is explained by sequence motif composition

We reasoned that the ancestral (consensus) sequence of RSINE1 might explain its propensity to bind CRs and promote the emergence of circadian enhancers. RSINE1 is a tRNA^Lys^-derived SINE family that belongs to the B4 superfamily, and its closest family relatives in the mouse genome are the B4 and B4A families [40, 79, 80]. While some other members of the B4 superfamily are significantly enriched to varying degrees in CR binding sites, none shows levels of enrichment comparable to that of RSINE1 (Fig. 2B). Thus, we hypothesized that RSINE1 may have unique sequence motifs that predispose these elements to recruit CRs. To test this idea, we compared the consensus sequences of RSINE1, B4, and B4A obtained from Repbase [74] for the presence of E-box motifs (CACGTG), which are recognized by CLOCK and BMAL1, as well as RORE motifs (RGGTA), which recruit nuclear receptors (NRs). NRs are known to be important co-regulators of circadian gene expression in the liver and include RORα [81], REV-ERBs [82], and LXR/RXR [83], which have well-characterized roles in liver metabolism [71] and circadian transcriptional regulation [57, 66]. We also searched for D-box motifs, which are bound by TEF, HLF, and DBP, three additional circadian TFs that orchestrate rhythmic transcription in different phases [57].

**Fig. 4:**
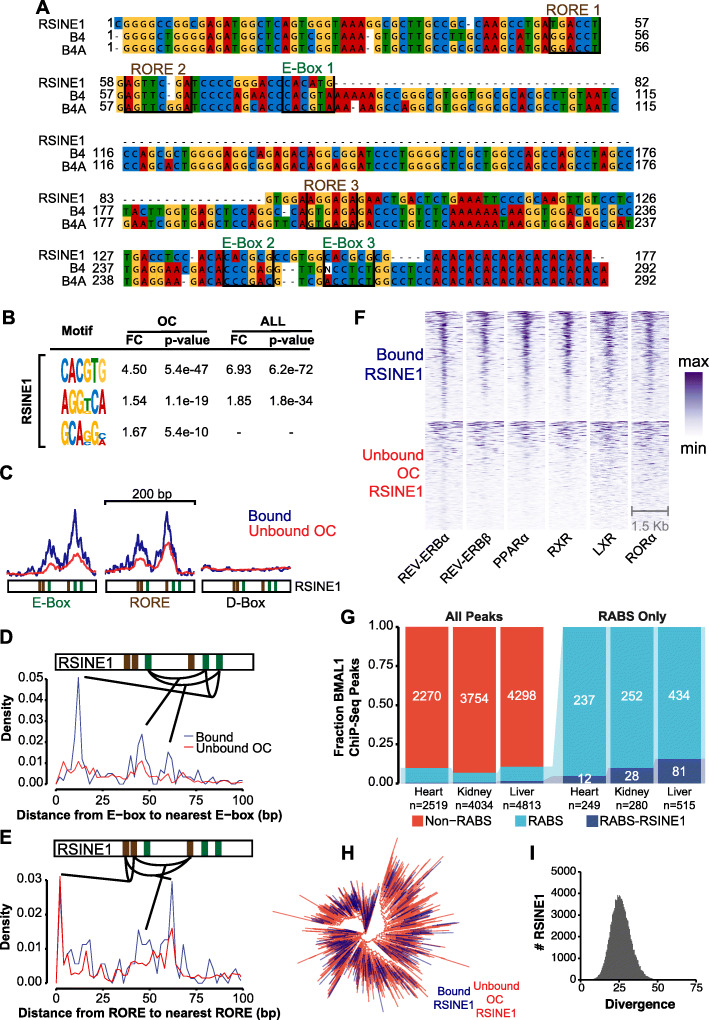
RSINE1 elements contain unique motif composition. **A** Multiple sequence alignment of B4 family SINES. RORE and E-Box pre-motifs are highlighted. **B** Motifs enriched in bound RSINE1 elements relative to unbound RSINE1 elements which reside in open chromatin (OC), or relative to all RSINE1 elements (ALL). The motif enrichment calculation was also performed using 500 bp of flanking sequences. **C** Density of D-Box (TTATGYAA), E-Box (CACRTG), and RORE (RGGTCA) motifs within bound (blue line; n = 328) and unbound OC (red line; n = 4279) classes of RSINE1 elements. Plots are centered on RSINE1s and extended 100 bp in either direction. **D** Average density of the distance from each E-Box and the next closest E-Box in bound RSINE1 elements (n = 328) and unbound open chromatin RSINE1 elements (n = 4279). **E** As in **D**, but density of the distance from each RORE to the next closest RORE motif. **F** ChIP-seq signal strength for various NRs centered at CR-bound RSINE1 elements (n = 328) or a randomly selected subset of unbound OC RSINE1 elements (n = 328). Sort order is maintained across columns; color is scaled relative to min-to-max for each individual factor (column). **G** Fraction of BMAL1 ChIP-seq peaks in the mouse heart, kidney, and liver which are (RABS) or are not (Non-RABS) associated with repetitive elements by > 50% overlap (left panel). Fraction of RABS which are or are not associated with RSINE1 elements (right panel) in each tissue. **H** Maximum likelihood tree of all CR-bound RSINE1 elements (n = 328) and a randomly selected set of unbound RSINE1 elements (n = 1000) (see the “Methods” section). **I** Histogram of divergence from the consensus of all RSINE1 insertions (Kimura 2-parameter)

We identified three E-Box motifs and two RORE motifs in the RSINE1 consensus sequence (Fig. 4A). All of the E-box motifs and two of the three RORE motifs present were not perfect matches to the empirically determined, optimal motifs for binding of CRs and NRs. Hereafter, we call these imperfect motifs “proto-motifs.” A pair of closely spaced (6-7 nt) E-Box motifs (CACGTG) is the optimal CLOCK:BMAL1 heterodimer binding site [59]. We observed one E-Box that is a single nucleotide change (CACATG to CACGTG; E-Box 1; Fig. 4A) away from the optimal sequence in the center of the RSINE1 consensus sequence; however, this motif is less likely to contribute significant regulatory activity as it is not a tandem motif, and similar sequences were present in B4 and B4A consensus sequences. Near the end of the RSINE1 consensus sequence, there were two E-Box motifs separated by 6 nt that are each a single nucleotide change away from the optimal sequence (CACGCG to CACGTG; E-Box 2 & 3; Fig. 4A). These two ancestral motifs each would require a single C-to-T mutation—a common deamination mutation in methylated DNA—to match the preferred CLOCK:BMAL1 binding site. The first RORE motif in the RSINE1 consensus is a perfect antisense motif and is closely followed by a sense motif that is two nucleotides away from an optimal RORE motif. However, these motifs are also present in the B4 and B4A consensus sequences. Together, these observations led us to conclude that E-Boxes 2 & 3 are the unique property of RSINE1 which most likely predisposed it to circadian regulatory activity.

We next sought to understand why a subset of RSINE1 copies in the genome are bound by CRs while most are not. We searched for motifs enriched in CR-bound RSINE1 elements with respect to OC RSINE1s and all RSINE1s. Discriminative unbiased motif discovery returned CACGTG and AGGKCA as enriched in bound RSINE1 elements with respect to unbound RSINE1 elements. Strikingly, these motifs correspond to E-Box and RORE elements respectively (Fig. 4B). To further explore the motif composition of these elements, we plotted profiles of E-Box and RORE motif occurrence weighted by motif confidence over 200-bp regions around the center of bound and OC unbound RSINE1s (Fig. 4C). Bound and unbound fractions both displayed characteristic patterns of tandem E-Box and RORE motifs, but few D-Box motifs. Notably, the summary plot revealed that while the spatial pattern is similar, the bound fraction has higher signal strength, which indicated that bound RSINE1 elements more frequently contain motifs that more closely match the optimal sequence than unbound OC RSINE1 elements.

We next examined the density distribution of distance from each optimal E-Box motif and the next closest E-box motif in order to identify characteristic spacing, which might better explain the difference between bound and unbound RSINE1 elements. Strikingly, the bound class had a strong enrichment for an E-Box ~ 12 bp from the nearest optimal E-Box, which was not present in unbound OC RSINE1 elements (Fig. 4D) and coincides with the pair of proto-motifs at the 3′ end of the consensus (Fig. 4A). When we examined the distance from RORE to RORE, we observed enrichment of closely adjacent motifs in both classes of RSINE1 elements (Fig. 4E). These results are consistent with the spacing of proto-motifs in the RSINE1 consensus and support the model that the bound class of RSINE1s gained their regulatory activity after transposition via maturation of imperfect CR/NR binding sites.

To verify that identified RORE elements are actually bound by NRs in the mouse liver, we turned to published ChIP-seq data for REV-ERBs [71], RXR, LXR, PPARα [70], and RORα [69] (Table 1). These datasets revealed that bound RSINE1 elements were occupied by the aforementioned NRs and that different NRs were bound simultaneously at the same RSINE1 elements, indicating that different NRs and/or CRs could be binding to these elements cooperatively. A randomly selected subset of unbound RSINE1s in open chromatin also displayed some NR binding but not to the degree of CR-bound RSINE1s (Fig. 4F). We reasoned that because NRs are liver-specific circadian co-regulators, the interplay of these CR/NR binding sites in the RSINE1 consensus may have given rise to tissue-specific regulatory elements. We then leveraged a recent analysis of tissue-specific BMAL1 binding sites using ChIP-seq in the mouse liver, heart, and kidney [72] and found that while the fraction of peaks overlapping with repetitive elements was relatively consistent, the fraction overlapping with RSINE1 elements was highest in the liver and markedly lower in other tissues (Fig. 4G). These results suggest that the majority of RSINE1-derived CR binding sites are liver-specific.

To rule out the possibility that the E-Box and RORE motifs enriched in bound RSINE1s could have emerged from the expansion of a slightly different progenitor containing pre-existing optimal motifs but forming a distinct RSINE1 subfamily, we aligned all CR-bound RSINE1 elements located in open chromatin with 1000 randomly selected RSINE1 copies and performed a phylogenetic analysis, the resulting tree had star-like topology with no subfamily structure coinciding with the two categories (Fig. 4H). This confirms that both bound and unbound RSINE1 elements descend from the same ancestral sequence which amplified rapidly throughout the genome, which is also supported by the normally distributed divergence observed for all RSINE1 insertions (Fig. 4I). This also validates our use of the consensus sequence as a proxy for the RSINE1 ancestral sequence, which could be skewed by the youngest RSINE1 elements if there had been multiple bursts. These results led us to conclude that RSINE1 spread imperfect CR/NR binding sites throughout the mouse genome, providing fodder for the evolution of new circadian enhancers rather than spreading “ready-made” enhancer modules.

### Circadian enhancer evolution from RSINE1 elements is context-dependent and lineage-specific

**Fig. 5:**
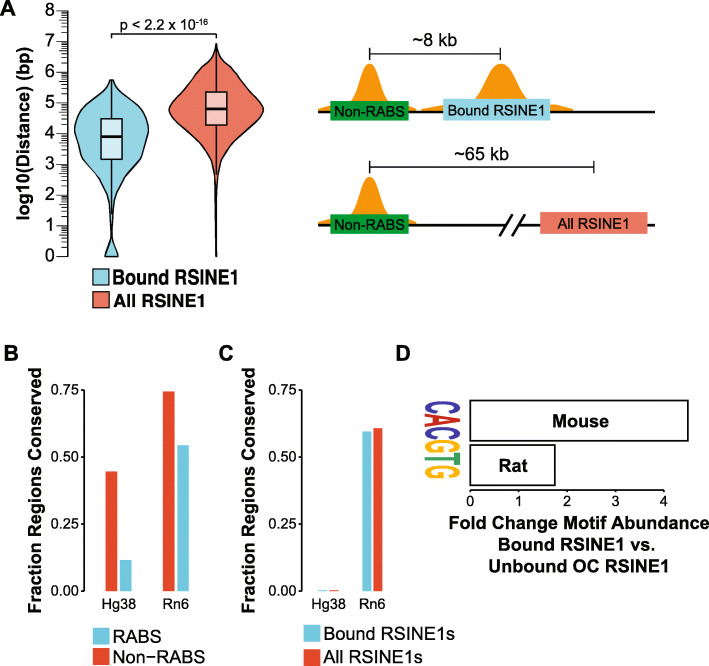
Evolution of CR binding sites from RSINE1 is context-dependent. **A** Distribution of distance from each CR-bound (n = 328) or all (n = 115,806) RSINE1 elements to the nearest non-repetitive CR binding site (Non-RABS). Boxplots show the median (center line), the 1st and 3rd quartiles (hinges), and 1.5 × IQR (whiskers). Statistical testing was done using the Mann-Whitney/Wilcoxon rank sum test. Distances on the schematized drawing represent the median distance in each class. **B** Fraction of Non-RABS and RABS CR binding sites that can be traced to the human or rat genomes using liftOver. **C** Fraction of CR-bound and unbound RSINE1 elements that can be traced to the human or rat genomes using liftOver. **D** Fold change CACGTG motifs detected in bound RSINE1 elements compared to unbound OC RSINE1 elements in the mouse as well as in syntenic orthologs in the rat

We next asked if the genomic location where RSINE1 inserted influences their propensity to act as circadian regulatory elements. More specifically, we asked whether the presence of an existing CR-bound sequence might favor the emergence of CR binding at RSINE1 elements. We determined the distance from each bound RSINE1 to the nearest non-repeat-derived CR binding site and compared this to the expected distance using the same calculation but using all genomic copies of RSINE1. Bound RSINE1s are significantly closer to Non-RABS (median ≈ 8 kb) than expected given the genomic distribution of all RSINE1 elements (median ≈ 65 kb; Fig. 5A). To determine whether these Non-RABS existed prior to RSINE1 insertion and thus shaped the evolutionary trajectory of RSINE1, we then queried whether Non-RABS CR binding sites have deeper phylogenetic roots than those in associated with repetitive elements by determining the fraction of regions for which an orthologous region could be identified in the rat and human genomes using liftOver [84]. We found that ~ 46% of the Non-RABS ChIP-seq peaks could be traced to orthologous regions in the human genome, and ~ 74% to the rat genome (Fig. 5B). This percentage is higher than the overall percentage of the mouse genome (~ 40%) that can be aligned confidently at the nucleotide level with the human genome [40], indicating that Non-RABS tend to be deeply conserved. Consistent with RSINE1’s murine specificity, ~ 60% of bound RSINE1 elements could be traced to an orthologous region in the rat genome, but virtually none (0.3%) was detected in the human genome (Fig. 5C). Thus, in general, RSINE1 derived CR binding sites are evolutionarily younger than Non-RABS and must have inserted nearby existing CR binding sites prior to their maturation into circadian enhancers. Given our previous finding that RSINE1 elements contained proto-motifs prior to their expansion, this result suggests that RSINE1 elements preferentially matured into optimal circadian regulatory elements after insertion nearby existing circadian regulatory sequences.

RSINE1 elements appear to have distributed imperfect CR/NR binding sites prior to the divergence of the mouse and rat lineages, and we hypothesized that some matured into lineage-specific circadian regulatory elements, i.e. after the divergence of these two species. To test this, we queried syntenic orthologs of mouse bound and unbound OC RSINE1 elements in the rat and performed motif discovery. This revealed that most elements which have developed perfect E-Box motifs (CACGTG) in the mouse have not developed the same motif in the rat (Fig. 5D). This indicates that bound RSINE1 elements in the mouse represent a subclass of RSINE1 elements which gained CR binding after the divergence of mouse and rat and thus are lineage-specific CR binding sites. Together, these findings show that the evolution of circadian regulatory elements from the proto-motif content of RSINE1 is a process which is context-dependent and lineage-specific.

### A minority of RSINE1 elements have matured into circadian enhancers

The above data indicate that RSINE1 elements are poised for maturation into circadian regulatory elements. We next searched for evidence for circadian enhancer activity of RSINE1 elements. A published study [69] used GRO-seq (Table 1) to measure nascent transcription over circadian time in the mouse liver and identified enhancer RNAs (eRNAs) that transcriptionally oscillate. We intersected our set of CR-bound RSINE1 elements with the coordinates of these oscillating eRNAs and identified 37 RSINE1 elements (11.3% of bound RSINE1s) for which 50% of the RSINE1 element falls within the coordinates of an oscillatory eRNA. One such example is an RSINE1 element that falls within the last intron of *Paip1* and is ~ 6 kb upstream of the start of *4833420G17Rik*, a gene with no identified function, which is in turn ~ 2 kb upstream of the TSS of *Tmem267*, a gene with a CR-bound promoter (Additional file 1: Fig. S6A). Furthermore, an independent study [63] identified *4833420G17Rik* as transcriptionally oscillating using Nascent-seq, and the RSINE1 element also overlapped with a high confidence BMAL1:CLOCK binding site designated as associated with rhythmic in phase transcription. Upon further inspection, we observed that the RSINE1 element had a characteristic pattern of oscillating CR binding and chromatin accessibility and was bound by NRs (Additional file 1: Fig. S6A). When we examined the sequence composition of this RSINE1, we observed two E-Box motifs at the end of the element and a RORE motif in the center. A D-Box motif was also present slightly upstream of the element, indicating that this RSINE1 could have matured into a bona fide circadian enhancer by virtue of the genomic context it inserted into. Another RSINE1 that falls within an oscillatory eRNA but does not have a D-Box in the flanking sequence lies immediately upstream of the promoter of *Aaed1* (Additional file 1: Fig. S6B).

**Fig. 6:**
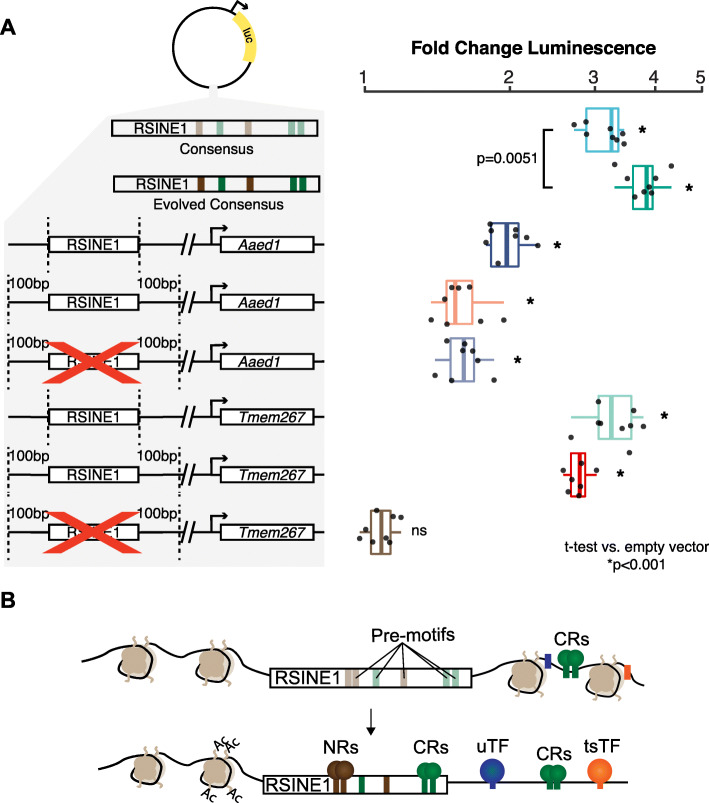
RSINE1 elements have enhancer activity in vitro. **A** Fold change luminescence when compared to an empty vector control after normalization to *Renilla* luciferase signal of enhancer reporter plasmids with various RSINE1-derived sequences inserted downstream of the luciferase gene. Included are RSINE1 consensus as is or with “evolved” E-Box and RORE motifs at highlighted locations (all motifs were changed to the canonical motif with no other changes), an RSINE1 element upstream of the promoter of *Aaed1* (see Additional file 1: Fig. S6B), and an RSINE1 upstream of the promoter of *4833420G17Rik* and *Tmem267* (see Additional file 1: Fig. S6A). *Tmem267* and *Aaed1* RSINE1s were cloned as the RSINE1 alone, with 100 bp of flanking sequence, and with the RSINE1 deleted from the flanking region as a reconstruction of the ancestral state. Experiments are representative of two biological transfection replicates with four technical replicates each. Significance was assessed using pairwise two-tailed t-tests compared to the empty vector. **B** A model of how RSINE1 has fine-tuned the circadian regulatory landscape of the murine lineage by distributing imperfect CR/NR binding sites with moderate regulatory activities that may modulate the activity of existing nearby circadian regulatory elements. NRs, nuclear receptors; CRs, circadian regulators; uTF, ubiquitous TF; tsTF, tissue-specific TF

We next used in vitro luciferase assays in Hepa 1-6 cells, a mouse hepatoma-derived cell line, to measure the enhancer activity of selected RSINE1 sequences. Importantly, we verified that CLOCK and BMAL1 are constitutively expressed in this cell type using public RNA-seq data (Table 1). We initially compared the consensus sequence from Repbase [74] to an “*in silico* evolved” sequence that we subjected to a total of 7 nucleotide substitutions to perfect the degenerate motifs in the RSINE1 consensus. We observed that the consensus alone had moderate in vitro enhancer activity, and the “evolved” version’s activity was significantly boosted (Fig. 6A). Next, we examined the two individual RSINE1 loci described above (*Tmem267* and *Aaed1*). For each, we tested the regulatory activity of the RSINE1 element alone, the RSINE1 with 100-bp flanking sequences, and a proxy for the ancestral pre-insertion locus where the RSINE1 was removed leaving only the flanking sequences joined at the insertion site. In both cases, we found that the RSINE1 element alone had the highest activity and that this activity was slightly lower when the flanking sequences were included (Fig. 6A). Removing the RSINE1 had no effect on the activity of the flanking sequences in the *Aaed1* locus reporter, but completely abolished the luciferase signal of the *Tmem267* locus. This pattern was consistent with the presence of a D-Box motif in the upstream flank of the *Tmem267*, which is known to be bound by the transcriptional repressor E4BP3 (also called NFIL3) as part of a secondary circadian transcriptional feedback loop with opposing phase to the classic CLOCK:BMAL feedback loop [85]. The most likely explanation for the phenomenon observed at the *Aaed1* RSINE1 locus is that the flanking sequence contains both activating and repressive TFBSs which in balance yields mild basal enhancer activity, and while the RSINE1 element contains stronger regulatory activity on its own, its presence in the flanking sequence does not disrupt this balance in a manner sufficient to alter enhancer activity. Take together, these assays illustrate how RSINE1 may have shaped the circadian regulatory landscape of the murine lineage (Fig. 6B) and highlight how the genomic context of each insertion of a family of repetitive elements could dictate which copies gain TF binding and regulatory activity in unique ways.

## Discussion

In this study, we show that a subset of CR binding sites in the mouse liver map within repetitive elements. This class had patterns of oscillatory CR binding that were indistinguishable from their non-repeat-derived binding sites, but characteristics consistent with their function as bona fide circadian enhancers were generally weaker than those observed for non-repeat-derived binding sites. This is in line with recent observations that only a small subset of TEs which display potential enhancer marks in mouse embryonic stem cells trigger gene expression changes when experimentally perturbed [86]. Furthermore, GO analysis of genes associated with TE-derived CR binding events show enrichment for genes linked to morphological phenotypes, including non-pigmented tail tip and variable body spotting, which are likely to be intra-specifically variable. We speculate that these associations reflect *cis*-regulatory connections that are evolutionarily young and might drive phenotypic plasticity akin to what is seen at the *Agouti* gene in mice, where the differential regulatory activity of IAP individuals leads to coat color variation [87–89]. However, we have not functionally demonstrated that RSINE1 elements drive cyclic transcription in vivo, and as a result, further study is needed to evaluate whether CR-bound TEs have directly contributed to changes in circadian gene expression and phenotypic variation.

### Seeding and maturation of TF proto-motifs by transposition

In this study, we examined the properties that predispose one family of TEs, RSINE1, to be bound by CRs and to acquire the biochemical hallmarks of circadian enhancers. We focused on the RSINE1 family because it was the only TE family in the mouse genome that was significantly overrepresented for CR binding events across all ChIP-seq datasets examined, suggesting that this family was in some way predisposed to be bound by CR. Furthermore, we observed that many of the CR-bound RSINE1 were also bound by NRs which are important regulators of circadian gene expression specifically in the liver [90, 91]. In addition to being bound by CRs and NRs, a small subset of these RSINE1 elements displayed features characteristic of circadian enhancers, including oscillatory H3K27 acetylation, DNase accessibility, and nascent transcription of eRNAs. We found that the recurrent binding of CRs and NRs to RSINE1 elements can be explained in part by the presence of sequence motifs in their ancestral sequence that are close, but imperfect match to the E-Box and RORE motifs that recruit CR and NR, respectively. In particular, a feature unique to RSINE1 when compared to other closely related SINE families is a pair of imperfect E-Box motifs at the 3′ end of the RSINE1 consensus sequence. Thus, unlike other cases of TE families enriched for TF binding events [20, 45, 48, 50, 51, 92, 93], RSINE1 did not introduce perfect TFBSs upon integration, but proto-motifs that repeatedly matured into optimal TFBSs within individual TE loci after their genomic integration.

Our analysis indicates that recurrent C-to-T mutations within the two proto E-boxes located at the 3′ end of RSINE1 represent the primary mutational path by which these elements acquired canonical CR binding sites. Interestingly, the C-to-T transition required within each of the proto-motif is at a CpG site (CACGCG-> CACGTG), which in mammalian genomes is ~ 10 times more likely to occur compared to mutations at non-CpG sites because of spontaneous deamination of methylated cytosine [94–96]. Because SINEs are heavily methylated in the mouse germline [97–99], it is likely that deamination at methylated CpG sites accelerated the maturation of the proto E-boxes residing within RSINE1 elements. The birth of TFBSs via CpG deamination in TEs has been observed previously in human Alu SINEs, which harbor proto-motifs for p53, PAX-6, and Myc that frequently mutate via CpG deamination to introduce new binding sites for these factors [100, 101]. Interestingly, Myc also binds an E-Box motif, though the proto-motif in the Alu consensus is CGCGCG, and thus requires two C-to-T transitions to mature into the canonical CACGTG motif. Our findings bolster the proposal that CpG deamination-mediated evolution of TFBSs from TE substrates is a pervasive force driving the emergence of new regulatory elements [100, 101]. This may be especially common for motifs such as the E-box for which many possible configurations of proto-motifs can generate a canonical motif (CACGTG) via CpG deamination. This is notable because the E-box is the binding motif of multiple TFs with diverse physiological or developmental functions, including CLOCK:BMAL1, but also MYC, and MYOD.

### Genomic environment of TE insertions influences their regulatory trajectory

Our study of RSINE1 underscores the role of the *cis*-regulatory context surrounding a TE insertion site in shaping the *cis*-regulatory potential of that TE. Our analyses indicate that RSINE1 elements that inserted nearby existing CR binding sites were predisposed to acquire CR binding activity upon maturation of their E-Box proto-motifs. This observation suggests a scenario whereby the insertion of RSINE1 near pre-existing CR binding sites determined, at least in part, whether they would become bound by CRs. In other words, the genomic regions where CRs were already binding at the time of RSINE1 amplification represented a favorable environment for the recruitment of CR to RSINE1 inserted in these regions. It is also possible that RSINE1s were only permitted to develop *bona fide* CR/NR binding sites when their insertion location allowed this gain of activity to fine-tune, replace, or become redundant with existing circadian regulatory elements, while the introduction of such regulatory activity at other genomic locations might have been deleterious. Our reporter assays are consistent with the notion that the genomic sequences flanking CR-bound RSINE1 loci can temper their *cis*-regulatory activity. This is in line with the idea that RSINE1 elements that were permitted to mature into CR enhancers did so preferentially when inserted in genomic regions that buffered the deleterious potential of aberrant circadian regulatory activity.

Our observation that RSINE1 elements preferentially gained CR binding activity when located nearby existing CR BS is reminiscent of a previously proposed model dubbed “epistatic capture” [102]. The authors reported that the combination of a MER20 and MER39 transposons forms the enhancer and promoter of decidual prolactin in humans. By tracing the evolutionary history of these two transposon insertions, they demonstrated that the MER39 element inserted nearby the pre-existing MER20 element, after which 7 nucleotide substitutions added 4 TFBSs which together drove elevated expression of prolactin. The authors argue that the presence of nearby TFBSs caused the stabilization of motifs and maturation of proto-motifs residing within the MER39 element [102]. Our findings for RSINE1 are in agreement with this model and suggest that the *cis*-regulatory landscape surrounding the insertion site of a TE profoundly influences its mutational path and *cis*-regulatory trajectory.

An important implication of the model outlined above is that it may also promote the turnover of TFBSs and *cis*-regulatory elements during evolution. Our data for RSINE1 suggest that RSINE1 are more likely to acquire a CR BS when they inserted near an existing CR BS. This process, in turn, would lead to redundancy in CR binding, which in some cases might lead to the mutational decay of the ancestral binding site, or be preserved by natural selection as a “buffer” against harmful *cis*-regulatory variation [103]. Choudhary and colleagues recently reported an elegant demonstration of redundant TFBSs introduced by transposition [104]. The authors show that TE-derived CTCF sites inserted nearby existing CTCF sites in human and mouse reinforce and maintain conserved higher order chromatin structure by conferring redundancy to conserved CTCF sites. The motif gain we see in RSINE1 insertions nearby existing CR BS could represent binding site turnover events. It is difficult to rigorously assess this without information regarding BMAL1 binding in the liver of other species, but this is an idea worthy of further investigation.

## Conclusions

In summary, we propose that the evolution of circadian regulatory elements from RSINE1 occurred through the following trajectory: (a) RSINE1 expanded in a single burst of rapid amplification in the murine lineage prior to the divergence of the mouse and the rat; (b) depending on the genomic context, most fixed RSINE1 insertions were neutral or mildly deleterious due to their weak regulatory activity; and (c) a small subset of elements were tolerated or adaptive due to their proximity to existing circadian regulatory elements and may have been co-opted as circadian enhancers either through binding site turnover, introduction of regulatory redundancy and buffering, or rarely could have wired a new gene into the circadian regulatory network (Fig. 6B).

Our study of the gradual emergence of *cis*-regulatory activity within RSINE1 suggests an alternative to the copy-and-paste model of TE co-option which stipulates that a TE inserts a ready-made enhancer module in a location where that activity becomes immediately beneficial [51, 105–107]. While this is an attractive model, such cases are likely to be rare because TEs carrying strongly active *cis*-regulatory modules, such as those often found within the long terminal repeats of retrotransposons and endogenous retroviruses, are substantially more likely to have deleterious effects upon insertion than they are to be adaptive. Instead, TEs with little to no intrinsic *cis*-regulatory activity upon insertion are more likely to be tolerated near genes and existing *cis*-regulatory elements. The “low regulatory profile” of these TEs might enable them to achieve higher copy number and disperse, on a large scale, proto-motifs poised for the emergence of new *cis*-regulatory elements. SINEs make excellent candidates for this model because they are short, noncoding, and typically derived from tRNA and other Pol III transcripts, thereby reducing their capacity to interfere with Pol II-regulation. Perhaps thanks to this property, SINEs have attained enormous copy numbers in virtually all mammalian genomes and they accumulate closer to genes than other types of TEs such as LINEs or ERVs [39, 40, 50, 108]. It may not be coincidental that many studies have identified ancient SINEs co-opted as enhancers and other complex *cis*-regulatory elements [109–116], but it has been difficult to discern features of these elements that promoted their exaptation. We speculate that we have caught RSINE1 at an early stage of the co-option process and that the principles uncovered herein illuminate how seemingly benign TEs like SINEs have been a pervasive source of *cis*-regulatory elements in vertebrate evolution.

## Methods

### Reanalysis of sequencing data

Fastq files were downloaded from SRA (Table 1). Reads were trimmed and filtered with cutadapt [117] (arguments: -a AGATCGGAAGAGCACACGTCTGAACTCCAGTCAC; -A AGATCGGAAGAGCGTCGTGTAGGGAAAGAGTGTAGATCTCGGTGGTCGCCGTATCATT; --minimum-length = 25), and aligned to the mm10 reference genome using bowtie2 [118] in paired-end mode where applicable (arguments: --local; --very-sensitive-local; --no-unal; --no-mixed; --no-discordant; -I 10; -X 1000). SOLiD reads were aligned using bowtie [119] in colorspace (arguments: -C; -m 1). Alignment files were processed, sorted, and filtered for uniquely mapping reads (q > 1) using samtools [120] and PCR duplicate reads removed with Picard MarkDuplicates. Peaks were called using macs2 [121] (arguments: -g mm; --call-summits).

### Genomic interval analyses

All intersections of sets of genomic intervals were performed using bedtools intersect [122] (arguments: -wo; -f 0.5), which requires that 50% of a feature in the query file overlaps a feature in the subject file in order to record an intersection. Non-intersecting regions were separately determined (arguments: -v; -f 0.5). Sets of CR ChIP-seq peak coordinates were intersected as queries against the output of RepeatMasker [75], which was retrieved from the UCSC genome browser and filtered to remove low complexity regions and simple repeats. All calculations of distances between genomic intervals were calculated using bedtools closest (arguments: -d).

### Expression profiles and heatmaps

Normalized signal coverage files were generated using DeepTools bamCoverage [123] (arguments: --binSize 10; --normalizeTo1x 2150570000). Single end reads were extended to match the average library insert fragment size reported at publication; empirically determined fragment size was used when paired-end sequence data was available. The scaling normalization employed here presents coverage in each bin as a ratio of observed coverage to expected genome-wide coverage assuming equal distribution of reads (1x coverage) and accounts for the mappable size of the genome, read depth, and read length. Profile meta-plots and heatmaps were generated using DeepTools [123]. Coverage matrixes were generated using computeMatrix in reference-point mode (arguments: --referencePoint center; --missingDataAsZero). Heatmaps were created with plotHeatmap and profiles were created with plotProfile (arguments: --plotType se). Quantification of signal in peaks was performed by using bigWigSummary to extract mean coverage at each genomic interval, followed by statistical testing in R using the packages kruskal.test and dunn.test. For visualization purposes, when plotting heatmaps designed to compare between sets of intervals with different sample sizes (for example, RABS-RSINE1 n = 328 and Non-RABS-RSINE1 n = 115,806), the larger set of intervals was subsampled to match the smallest set of intervals prior to matrix computation.

### Gene Ontology analysis

Enrichment of mouse phenotypes with each group of genomic intervals was calculated by GREAT [77] using the two nearest genes within 1 Mb of each E-Box motif.

### Repeat element enrichment

Enrichment was calculated as described [78] (https://github.com/4ureliek/TEanalysis) by shuffling TEs such that the distance to the nearest TSS and number of elements on each chromosome was maintained. These shuffled TEs were intersected with the mm10 RepeatMasker annotations [75] to determine the number of expected intersections from each TE and TE family. This procedure was bootstrapped 1000 times. A binomial test was then performed to determine significance, comparing the average expectation to the observed value.

### Consensus alignment

Consensus sequences of RSINE1, B4, and B4A were obtained from Repbase [74] and aligned using Clustal Omega [124]. The resulting alignment was visualized and manually curated using Jalview [125]. Relevant motifs were identified manually.

### Motif analysis

Sequences of bound, OC unbound, and all RSINE1 elements were retrieved using bedtools getfasta [122]. Unbiased motif discovery was performed using DREME [126] with bound RSINE1s as primary sequences, and both OC unbound and all RSINE1s as control sequences, sequentially. This procedure was repeated using the 500-bp flanks (left and right) of each RSINE1 element in each set. Locations of E-box (CACRTG), RORE (RGGTCA), and D-Box (TTATGYAA) motifs were predicted genome wide using FIMO [127] (arguments: --thresh 0.01; --max-stored-scores 10000000). Bedtools merge [122] was used to merge overlapping motifs and average the FIMO score over the merged region, and the utility bedGraphToBigWig was used to generate coverage tracks where the coverage represents genome-wide motif occurrence weighted by score. Heatmaps were generated as above. The distance to the nearest E-Box motif from each set of intervals (peaks and repeats) was calculated using bedtools closest [122] (arguments: -d), and E-Boxes which fall within the boundaries of the peak (and therefore are distance = 0) were retained. For analysis of distance between motifs, we filtered coordinates of predicted motifs by FIMO log-odds score, retaining only motifs with the highest possible score. Bedtools intersect (arguments: -f 1; -wa) reported motifs that completely overlap with a given set of repeats; the distance from these overlapping motifs to the nearest non-overlapping motif was calculated using bedtools closest (arguments: -io; -d).

### Conservation analysis

We attempted to map sets of genomic regions from the mm10 genome to the hg38 or rn6 genomes using liftOver (Kuhn et al. [84]) using appropriate chains and counted the fraction of intervals which did or did not lift over from each set of elements.

### RSINE1 phylogeny

We began with sequences of the 328 bound RSINE1 elements, as well as 1000 randomly selected elements from the set of all RSINE1s in the mm10 genome assembly (n = 115,806). We aligned these sequences using Clustal Omega [124] with default settings. From this alignment, we calculated a maximum likelihood tree with FastTree 2 [128] using default settings for nucleotide alignments. The tree was visualized with ggtree [129] and branches were colored by their designation as bound or unbound RSINE1 elements.

### Mouse/rat motif comparison

We started with the set of CR-bound RSINE1 elements (n = 328) and unbound elements residing in open chromatin (n = 4583) and attempted to assign syntenic orthologs of each element using liftOver [84]. We were able to find orthologous regions for 195 bound RSINE1 elements (59.5%) and 2662 unbound elements (58.1%). We queried the sequences of each of these elements using bedtools getfasta [122] from the mm10 and rn6 genome assemblies and used DREME [126] as above to find motifs enriched in orthologous bound RSINE1 elements when compared to unbound RSINE1 elements.

### Tissue-specific BMAL1 analysis

We started with coordinates of BMAL1 binding sites in the liver, kidney, and heart [72] in the mm10 genome assembly. We performed an intersection as described above (requiring 50% overlap of a given peak with a repetitive element to designate that peak as a RABS) between each of these sets of coordinates and the output of RepeatMasker [75] which was retrieved from the UCSC genome browser and filtered to remove low complexity regions and simple repeats. We counted the number of Non-RABS, RABS, and RSINE1-RABS and plotted the fraction each category represents with respect to the total set of peaks, and the fraction of RABS which are RSINE1-derived.

### Luciferase reporter assays

We accessed the RSINE1 consensus sequence from Repbase [74] and created an “*in silico* evolved” version by changing a total of 7 nt to “perfect” the E-Box and RORE motifs highlighted in Fig. 4A (RORE 1: unchanged; RORE 2: AGTTCG>AGGTCA; RORE 3: AGGAGA>AGGTCA; E-Box 1: CACATG>CACGTG; E-Box 2&3: CACGCG>CACGTG). We also accessed, from the mm10 genome assembly, the sequences of *Tmem267* and *Aaed1* RSINE1 elements highlighted in Additional file 1: Fig. S6, both with and without 100 nt of flanking sequence on the 5′ and 3′ ends. All of these sequences were synthesized as gBlocks® Gene Fragments (Integrated DNA Technologies, Coralville, IA, USA) and cloned into pGL3::PRO (Promega Corporation, Madison, WI, USA) downstream of the luciferase gene. To generate vectors of *Tmem267* and Aaed1 RSINE1 flanking sequence only, existing vectors were amplified by PCR using Q5 High-Fidelity DNA Polymerase (New England Biolabs, Ipswich, MA, USA) with primers designed to delete the RSINE1 element by site-directed mutagenesis. All vectors were validated by Sanger sequencing. Hepa 1-6 cells were obtained from ATCC and were subject to co-transfection with 500 ng of each of the constructs described above in conjunction with 500 ng pRL::SV40 Renilla Luciferase Control Vector (Promega Corporation, Madison, WI, USA) using Lipofectamine-2000 (Thermo Fisher Scientific, Waltham, MA, USA). After 18 h, firefly and *Renilla* luciferase luminescence was measured using the Dual-Glo® Luciferase Assay System (Promega Corporation, Madison, WI, USA). Firefly luminescence values were normalized to *Renilla* luminescence and compared to the empty pGL3::PRO vector.

## Supplementary Information

**Additional file 1.** Contains Supplementary Figures S1–S6.

**Additional file 2.** Review history.

## Data Availability

All data used in this study was previously published and accession numbers can be found in Table 1.

## References

[1] Carroll SB, Prud’homme B, Gompel N (2008). Regulating evolution. Sci Am..

[2] King MC, Wilson AC (1975). Evolution at two levels in humans and chimpanzees. Science..

[3] Wagner GP, Lynch VJ (2010). Evolutionary novelties. Curr Biol..

[4] Romero IG, Ruvinsky I, Gilad Y (2012). Comparative studies of gene expression and the evolution of gene regulation. Nat Rev Genet..

[5] Reilly SK, Noonan JP (2016). Evolution of gene regulation in humans. Annu Rev Genomics Hum Genet..

[6] Stern DL, Orgogozo V (2008). The loci of evolution: how predictable is genetic evolution?. Evolution (N Y)..

[7] Wray GA (2007). The evolutionary significance of cis-regulatory mutations. Nat Rev Genet..

[8] Rebeiz M, Tsiantis M (2017). Enhancer evolution and the origins of morphological novelty. Curr Opin Genet Dev..

[9] Pennacchio LA, Bickmore W, Dean A, Nobrega MA, Bejerano G (2013). Enhancers: five essential questions. Nat Rev Genet..

[10] Rubinstein M, de Souza FSJ (2013). Evolution of transcriptional enhancers and animal diversity. Philos Trans R Soc B Biol Sci..

[11] Villar D, Flicek P, Odom DT (2014). Evolution of transcription factor binding in metazoans-mechanisms and functional implications. Nat Rev Genet..

[12] Wittkopp PJ (2010). Variable transcription factor binding: a mechanism of evolutionary change. PLoS Biol..

[13] Levine M, Cattoglio C, Tjian R (2014). Looping back to leap forward: Transcription enters a new era. Cell..

[14] Schoenfelder S, Fraser P (2019). Long-range enhancer–promoter contacts in gene expression control. Nat Rev Genet..

[15] Arnold CD, Gerlach D, Stelzer C, Boryń ŁM, Rath M, Stark A (2013). Genome-wide quantitative enhancer activity maps identified by STARR-seq. Science..

[16] Heidari N, Phanstiel DH, He C, Grubert F, Jahanbani F, Kasowski M (2014). Genome-wide map of regulatory interactions in the human genome. Genome Res..

[17] Noonan JP (2009). Regulatory DNAs and the evolution of human development. Curr Opin Genet Dev..

[18] Blow MJ, McCulley DJ, Li Z, Zhang T, Akiyama JA, Holt A (2010). ChIP-seq identification of weakly conserved heart enhancers. Nat Genet..

[19] Cotney J, Leng J, Yin J, Reilly SK, Demare LE, Emera D (2013). The evolution of lineage-specific regulatory activities in the human embryonic limb. Cell..

[20] Kunarso G, Chia NY, Jeyakani J, Hwang C, Lu X, Chan YS (2010). Transposable elements have rewired the core regulatory network of human embryonic stem cells. Nat Genet..

[21] Schmidt D, Wilson MD, Ballester B, Schwalie PC, Brown GD, Marshall A (2010). Five-vertebrate ChlP-seq reveals the evolutionary dynamics of transcription factor binding. Science..

[22] Shen Y, Yue F, Mc Cleary DF, Ye Z, Edsall L, Kuan S (2012). A map of the cis-regulatory sequences in the mouse genome. Nature..

[23] Stefflova K, Thybert D, Wilson MD, Streeter I, Aleksic J, Karagianni P, Brazma A, Adams DJ, Talianidis I, Marioni JC, Flicek P, Odom DT (2013). Cooperativity and rapid evolution of cobound transcription factors in closely related mammals. Cell..

[24] Vierstra J, Rynes E, Sandstrom R, Zhang M, Canfield T, Scott Hansen R (2014). Mouse regulatory DNA landscapes reveal global principles of cis-regulatory evolution. Science..

[25] Douglas AT, Hill RE (2014). Variation in vertebrate Cis-regulatory elements in evolution and disease. Transcription..

[26] Long HK, Prescott SL, Wysocka J (2016). Ever-changing landscapes: transcriptional enhancers in development and evolution. Cell..

[27] Glinsky G, Barakat TS (2019). The evolution of Great Apes has shaped the functional enhancers’ landscape in human embryonic stem cells. Stem Cell Res..

[28] Villar D, Berthelot C, Aldridge S, Rayner TF, Lukk M, Pignatelli M (2015). Enhancer evolution across 20 mammalian species. Cell..

[29] Berthelot C, Villar D, Horvath JE, Odom DT, Flicek P (2018). Complexity and conservation of regulatory landscapes underlie evolutionary resilience of mammalian gene expression. Nat Ecol Evol..

[30] Dickel DE, Ypsilanti AR, Pla R, Zhu Y, Barozzi I, Mannion BJ (2018). Ultraconserved enhancers are required for normal development. Cell.

[31] Li S, Kvon EZ, Visel A, Pennacchio LA, Ovcharenko I (2019). Stable enhancers are active in development, and fragile enhancers are associated with evolutionary adaptation. Genome Biol..

[32] Eichenlaub MP, Ettwiller L (2011). De novo genesis of enhancers in vertebrates. Plos Biol..

[33] Klein JC, Keith A, Agarwal V, Durham T, Shendure J (2018). Functional characterization of enhancer evolution in the primate lineage. Genome Biol..

[34] Trizzino M, Park Y, Holsbach-Beltrame M, Aracena K, Mika K, Caliskan M (2017). Transposable elements are the primary source of novelty in primate gene regulation. Genome Res..

[35] Young RS (2016). Lineage-specific genomics: frequent birth and death in the human genome: the human genome contains many lineage-specific elements created by both sequence and functional turnover. BioEssays..

[36] Wells JN, Feschotte C (2020). A field guide to eukaryotic transposable elements. Annu Rev Genet.

[37] de Koning APJ, Gu W, Castoe TA, Batzer MA, Pollock DD (2011). Repetitive elements may comprise over two-thirds of the human genome. Plos Genet..

[38] Mikkelsen TS, Wakefield MJ, Aken B, Amemiya CT, Chang JL, Duke S (2007). Genome of the marsupial Monodelphis domestica reveals innovation in non-coding sequences. Nature..

[39] Lander ES, Linton LM, Birren B, Nusbaum C, Zody MC, Baldwin J, Devon K, Dewar K, Doyle M, FitzHugh W, Funke R, Gage D, Harris K, Heaford A, Howland J, Kann L, Lehoczky J, LeVine R, McEwan P, McKernan K, Meldrim J, Mesirov JP, Miranda C, Morris W, Naylor J, Raymond C, Rosetti M, Santos R, Sheridan A, Sougnez C, Stange-Thomann Y, Stojanovic N, Subramanian A, Wyman D, Rogers J, Sulston J, Ainscough R, Beck S, Bentley D, Burton J, Clee C, Carter N, Coulson A, Deadman R, Deloukas P, Dunham A, Dunham I, Durbin R, French L, Grafham D, Gregory S, Hubbard T, Humphray S, Hunt A, Jones M, Lloyd C, McMurray A, Matthews L, Mercer S, Milne S, Mullikin JC, Mungall A, Plumb R, Ross M, Shownkeen R, Sims S, Waterston RH, Wilson RK, Hillier LW, McPherson J, Marra MA, Mardis ER, Fulton LA, Chinwalla AT, Pepin KH, Gish WR, Chissoe SL, Wendl MC, Delehaunty KD, Miner TL, Delehaunty A, Kramer JB, Cook LL, Fulton RS, Johnson DL, Minx PJ, Clifton SW, Hawkins T, Branscomb E, Predki P, Richardson P, Wenning S, Slezak T, Doggett N, Cheng JF, Olsen A, Lucas S, Elkin C, Uberbacher E, Frazier M, Gibbs RA, Muzny DM, Scherer SE, Bouck JB, Sodergren EJ, Worley KC, Rives CM, Gorrell JH, Metzker ML, Naylor SL, Kucherlapati RS, Nelson DL, Weinstock GM, Sakaki Y, Fujiyama A, Hattori M, Yada T, Toyoda A, Itoh T, Kawagoe C, Watanabe H, Totoki Y, Taylor T, Weissenbach J, Heilig R, Saurin W, Artiguenave F, Brottier P, Bruls T, Pelletier E, Robert C, Wincker P, Smith DR, Doucette-Stamm L, Rubenfield M, Weinstock K, Lee HM, Dubois J, Rosenthal A, Platzer M, Nyakatura G, Taudien S, Rump A, Yang H, Yu J, Wang J, Huang G, Gu J, Hood L, Rowen L, Madan A, Qin S, Davis RW, Federspiel NA, Abola AP, Proctor MJ, Myers RM, Schmutz J, Dickson M, Grimwood J, Cox DR, Olson MV, Kaul R, Raymond C, Shimizu N, Kawasaki K, Minoshima S, Evans GA, Athanasiou M, Schultz R, Roe BA, Chen F, Pan H, Ramser J, Lehrach H, Reinhardt R, McCombie W, de la Bastide M, Dedhia N, Blöcker H, Hornischer K, Nordsiek G, Agarwala R, Aravind L, Bailey JA, Bateman A, Batzoglou S, Birney E, Bork P, Brown DG, Burge CB, Cerutti L, Chen HC, Church D, Clamp M, Copley RR, Doerks T, Eddy SR, Eichler EE, Furey TS, Galagan J, Gilbert JG, Harmon C, Hayashizaki Y, Haussler D, Hermjakob H, Hokamp K, Jang W, Johnson LS, Jones TA, Kasif S, Kaspryzk A, Kennedy S, Kent WJ, Kitts P, Koonin EV, Korf I, Kulp D, Lancet D, Lowe TM, McLysaght A, Mikkelsen T, Moran JV, Mulder N, Pollara VJ, Ponting CP, Schuler G, Schultz J, Slater G, Smit AF, Stupka E, Szustakowki J, Thierry-Mieg D, Thierry-Mieg J, Wagner L, Wallis J, Wheeler R, Williams A, Wolf YI, Wolfe KH, Yang SP, Yeh RF, Collins F, Guyer MS, Peterson J, Felsenfeld A, Wetterstrand KA, Patrinos A, Morgan MJ, de Jong P, Catanese JJ, Osoegawa K, Shizuya H, Choi S, Chen YJ, Szustakowki J, International Human Genome Sequencing Consortium (2001). Initial sequencing and analysis of the human genome. Nature..

[40] Waterston RH, Lindblad-Toh K, Birney E, Rogers J, Abril JF, Agarwal P (2002). Initial sequencing and comparative analysis of the mouse genome. Nature..

[41] Kapusta A, Suh A, Feschotte C (2017). Dynamics of genome size evolution in birds and mammals. Proc Natl Acad Sci..

[42] Sudmant PH, Rausch T, Gardner EJ, Handsaker RE, Abyzov A, Huddleston J (2015). An integrated map of structural variation in 2,504 human genomes. Nature..

[43] Feschotte C (2008). Transposable elements and the evolution of regulatory networks. Nat Rev Genet..

[44] Chuong EB, Elde NC, Feschotte C (2017). Regulatory activities of transposable elements: from conflicts to benefits. Nat Rev Genet..

[45] Sundaram V, Choudhary MNK, Pehrsson E, Xing X, Fiore C, Pandey M (2017). Functional cis-regulatory modules encoded by mouse-specific endogenous retrovirus. Nat Commun..

[46] Sundaram V, Wysocka J (2020). Transposable elements as a potent source of diverse cis-regulatory sequences in mammalian genomes. Philos Trans R Soc Lond B Biol Sci..

[47] Rebollo R, Romanish MT, Mager DL (2012). Transposable elements: an abundant and natural source of regulatory sequences for host genes. Annu Rev Genet..

[48] Sundaram V, Cheng Y, Ma Z, Li D, Xing X, Edge P, Snyder MP, Wang T (2014). Widespread contribution of transposable elements to the innovation of gene regulatory networks. Genome Res..

[49] Wang T, Zeng J, Lowe CB, Sellers RG, Salama SR, Yang M, Burgess SM, Brachmann RK, Haussler D (2007). Species-specific endogenous retroviruses shape the transcriptional network of the human tumor suppressor protein p53. Proc Natl Acad Sci..

[50] Schmidt D, Schwalie PC, Wilson MD, Ballester B, Gonalves Â, Kutter C (2012). Waves of retrotransposon expansion remodel genome organization and CTCF binding in multiple mammalian lineages. Cell..

[51] Chuong EB, Elde NC, Feschotte C (2016). Regulatory evolution of innate immunity through co-option of endogenous retroviruses. Science..

[52] Bourque G, Leong B, Vega VB, Chen X, Yen LL, Srinivasan KG (2008). Evolution of the mammalian transcription factor binding repertoire via transposable elements. Genome Res..

[53] Jacques P-É, Jeyakani J, Bourque G (2013). The majority of primate-specific regulatory sequences are derived from transposable elements. Plos Genet..

[54] Gibbs RA, Weinstock GM, Metzker ML, Muzny DM, Sodergren EJ, Scherer S (2004). Genome sequence of the Brown Norway rat yields insights into mammalian evolution. Nature..

[55] Lynch VJ, Leclerc RD, May G, Wagner GP (2011). Transposon-mediated rewiring of gene regulatory networks contributed to the evolution of pregnancy in mammals. Nat Genet..

[56] Mohawk JA, Green CB, Takahashi JS (2012). Central and peripheral circadian clocks in mammals. Annu Rev Neurosci..

[57] Takahashi JS (2017). Transcriptional architecture of the mammalian circadian clock. Nat Rev Genet..

[58] Welsh DK, Takahashi JS, Kay SA (2009). Suprachiasmatic nucleus: cell autonomy and network properties. Annu Rev Physiol..

[59] Rey G, Cesbron F, Rougemont J, Reinke H, Brunner M, Naef F (2011). Genome-wide and phase-specific DNA-binding rhythms of BMAL1 control circadian output functions in mouse liver. Plos Biol..

[60] Gekakis N, Staknis D, Nguyen HB, Davis FC, Wilsbacner LD, King DP (1998). Role of the CLOCK protein in the mammalian circadian mechanism. Science..

[61] Kume K, Zylka MJ, Sriram S, Shearman LP, Weaver DR, Jin X, Maywood ES, Hastings MH, Reppert SM (1999). mCRY1 and mCRY2 are essential components of the negative limb of the circadian clock feedback loop. Cell..

[62] Menet JS, Pescatore S, Rosbash M (2014). CLOCK: BMAL1 is a pioneer-like transcription factor. Genes Dev..

[63] Trott AJ, Menet JS (2018). Regulation of circadian clock transcriptional output by CLOCK:BMAL1. Plos Genet..

[64] Le Martelot G, Claudel T, Gatfield D, Schaad O, Kornmann B, Lo Sasso G (2009). REV-ERBα participates in circadian SREBP signaling and bile acid homeostasis. Plos Biol..

[65] Turek FW, Joshu C, Kohsaka A, Lin E, Ivanova G, McDearmon E (2005). Obesity and metabolic syndrome in circadian Clock mutant mice. Science..

[66] Zhao X, Cho H, Yu RT, Atkins AR, Downes M, Evans RM (2014). Nuclear receptors rock around the clock. EMBO Rep..

[67] Koike N, Yoo SH, Huang HC, Kumar V, Lee C, Kim TK (2012). Transcriptional architecture and chromatin landscape of the core circadian clock in mammals. Science..

[68] Sobel JA, Krier I, Andersin T, Raghav S, Canella D, Gilardi F, Kalantzi AS, Rey G, Weger B, Gachon F, Dal Peraro M, Hernandez N, Schibler U, Deplancke B, Naef F, CycliX consortium (2017). Transcriptional regulatory logic of the diurnal cycle in the mouse liver. Plos Biol..

[69] Fang B, Everett LJ, Jager J, Briggs E, Armour SM, Feng D, Roy A, Gerhart-Hines Z, Sun Z, Lazar MA (2014). Circadian enhancers coordinate multiple phases of rhythmic gene transcription in vivo. Cell..

[70] Boergesen M, Pedersen TÅ, Gross B, van Heeringen SJ, Hagenbeek D, Bindesbøll C (2012). Genome-wide profiling of liver X receptor, retinoid X receptor, and peroxisome proliferator-activated receptor α in mouse liver reveals extensive sharing of binding sites. Mol Cell Biol..

[71] Cho H, Zhao X, Hatori M, Yu RT, Barish GD, Lam MT, Chong LW, DiTacchio L, Atkins AR, Glass CK, Liddle C, Auwerx J, Downes M, Panda S, Evans RM (2012). Regulation of circadian behaviour and metabolism by REV-ERB-α and REV-ERB-β. Nature..

[72] Beytebiere JR, Trott AJ, Greenwell BJ, Osborne CA, Vitet H, Spence J (2019). Tissue-specific BMAL1 cistromes reveal that rhythmic transcription is associated with rhythmic enhancer–enhancer interactions. Genes Dev..

[73] Huang L, Damle SS, Booten S, Singh P, Sabripour M, Hsu J, Jo M, Katz M, Watt A, Hart CE, Freier SM, Monia BP, Guo S (2015). Partial hepatectomy induced long noncoding RNA inhibits hepatocyte proliferation during liver regeneration. Plos One..

[74] Jurka J, Kapitonov VV, Pavlicek A, Klonowski P, Kohany O, Walichiewicz J (2005). Repbase Update, a database of eukaryotic repetitive elements. Cytogenet Genome Res..

[75] Smit A, Hubley R, Green P (2013). RepeatMasker Open-4.0.

[76] Kent WJ, Sugnet CW, Furey TS, Roskin KM, Pringle TH, Zahler AM (2002). The Human Genome Browser at UCSC. Genome Res..

[77] McLean CY, Bristor D, Hiller M, Clarke SL, Schaar BT, Lowe CB (2010). GREAT improves functional interpretation of cis-regulatory regions. Nat Biotechnol..

[78] Kapusta A, Kronenberg Z, Lynch VJ, Zhuo X, Ramsay LA, Bourque G, Yandell M, Feschotte C (2013). Transposable elements are major contributors to the origin, diversification, and regulation of vertebrate long noncoding RNAs. Plos Genet..

[79] Hubley R, Finn RD, Clements J, Eddy SR, Jones TA, Bao W, Smit AFA, Wheeler TJ (2016). The Dfam database of repetitive DNA families. Nucleic Acids Res..

[80] Lee IY, Westaway D, Smit AFA, Wang K, Seto J, Chen L, Acharya C, Ankener M, Baskin D, Cooper C, Yao H, Prusiner SB, Hood LE (1998). Complete genomic sequence and analysis of the prion protein gene region from three mammalian species. Genome Res..

[81] Giguere V, Tini M, Flock G, Ong E, Evans RM, Otulakowski G (1994). Isoform-specific amino-terminal domains dictate DNA-binding properties of RORα, a novel family of orphan hormone nuclear receptors. Genes Dev..

[82] Guillaumond F, Dardente H, Giguère V, Cermakian N (2005). Differential control of Bmal1 circadian transcription by REV-ERB and ROR nuclear receptors. J Biol Rhythms..

[83] Shen Q, Bai Y, Chang KCN, Wang Y, Burris TP, Freedman LP (2011). Liver X receptor-retinoid X receptor (LXR-RXR) heterodimer cistrome reveals coordination of LXR and AP1 signaling in keratinocytes. J Biol Chem..

[84] Kuhn RM, Haussler D, James KW (2013). The UCSC genome browser and associated tools. Brief Bioinform..

[85] Mitsui S, Yamaguchi S, Matsuo T, Ishida Y, Okamura H (2001). Antagonistic role of E4BP4 and PAR proteins in the circadian oscillatory mechanism. Genes Dev..

[86] Todd CD, Deniz Ö, Taylor D, Branco MR (2019). Functional evaluation of transposable elements as enhancers in mouse embryonic and trophoblast stem cells. Elife..

[87] Duhl DMJ, Vrieling H, Miller KA, Wolff GL, Barsh GS (1994). Neomorphic agouti mutations in obese yellow mice. Nat Genet..

[88] Morgan HD, Sutherland HGE, Martin DIK, Whitelaw E (1999). Epigenetic inheritance at the agouti locus in the mouse. Nat Genet..

[89] Whitelaw E, Martin DIK (2001). Retrotransposons as epigenetic mediators of phenotypic variation in mammals. Nat Genet..

[90] Duez H, Staels B (2008). The nuclear receptors Rev-erbs and RORs integrate circadian rhythms and metabolism. Diabetes Vasc Dis Res..

[91] Solt LA, Kojetin DJ, Burris TP (2011). The REV-ERBs and RORs: molecular links between circadian rhythms and lipid homeostasis. Future Med Chem..

[92] Grow EJ, Flynn RA, Chavez SL, Bayless NL, Wossidlo M, Wesche DJ, Martin L, Ware CB, Blish CA, Chang HY, Reijo Pera RA, Wysocka J (2015). Intrinsic retroviral reactivation in human preimplantation embryos and pluripotent cells. Nature..

[93] Roman AC, Benitez DA, Carvajal-Gonzalez JM, Fernandez-Salguero PM (2008). Genome-wide B1 retrotransposon binds the transcription factors dioxin receptor and Slug and regulates gene expression in vivo. Proc Natl Acad Sci..

[94] Simmen MW (2008). Genome-scale relationships between cytosine methylation and dinucleotide abundances in animals. Genomics..

[95] Bird AP (1980). DNA methylation and the frequency of CpG in animal DNA. Nucleic Acids Res..

[96] Antequera F, Bird A (1993). Number of CpG islands and genes in human and mouse. Proc Natl Acad Sci..

[97] Ichiyanagi K, Li Y, Watanabe T, Ichiyanagi T, Fukuda K, Kitayama J (2011). Locus- and domain-dependent control of DNA methylation at mouse B1 retrotransposons during male germ cell development. Genome Res..

[98] Molaro A, Falciatori I, Hodges E, Aravin AA, Marran K, Rafii S, McCombie WR, Smith AD, Hannon GJ (2014). Two waves of de novo methylation during mouse germ cell development. Genes Dev..

[99] Popp C, Dean W, Feng S, Cokus SJ, Andrews S, Pellegrini M (2010). Genome-wide erasure of DNA methylation in mouse primordial germ cells is affected by AID deficiency. Nature..

[100] Zemojtel T, Kielbasa SM, Arndt PF, Behrens S, Bourque G, Vingron M (2011). CpG deamination creates transcription factor-binding sites with high efficiency. Genome Biol Evol..

[101] Zemojtel T, Kielbasa SM, Arndt PF, Chung HR, Vingron M (2009). Methylation and deamination of CpGs generate p53-binding sites on a genomic scale. Trends Genet..

[102] Emera D, Wagner GP (2012). Transformation of a transposon into a derived prolactin promoter with function during human pregnancy. Proc Natl Acad Sci..

[103] Cannavò E, Khoueiry P, Garfield DA, Geeleher P, Zichner T, Gustafson EH (2016). Shadow enhancers are pervasive features of developmental regulatory networks. Curr Biol..

[104] Choudhary MNK, Friedman RZ, Wang JT, Jang HS, Zhuo X, Wang T (2020). Co-opted transposons help perpetuate conserved higher-order chromosomal structures. Genome Biol..

[105] Dunn-Fletcher CE, Muglia LM, Pavlicev M, Wolf G, Sun M-A, Hu Y-C, Huffman E, Tumukuntala S, Thiele K, Mukherjee A, Zoubovsky S, Zhang X, Swaggart KA, Lamm KYB, Jones H, Macfarlan TS, Muglia LJ (2018). Anthropoid primate–specific retroviral element THE1B controls expression of CRH in placenta and alters gestation length. PLOS Biol..

[106] Chuong EB, Rumi MAK, Soares MJ, Baker JC (2013). Endogenous retroviruses function as species-specific enhancer elements in the placenta. Nat Genet..

[107] Pi W, Zhu X, Wu M, Wang Y, Fulzele S, Eroglu A, Ling J, Tuan D (2010). Long-range function of an intergenic retrotransposon. Proc Natl Acad Sci..

[108] Zhang Y, Romanish MT, Mager DL (2011). Distributions of transposable elements reveal hazardous zones in mammalian introns. PLoS Comput Biol..

[109] Notwell JH, Chung T, Heavner W, Bejerano G (2015). A family of transposable elements co-opted into developmental enhancers in the mouse neocortex. Nat Commun..

[110] Lowe CB, Bejerano G, Haussler D (2007). Thousands of human mobile element fragments undergo strong purifying selection near developmental genes. Proc Natl Acad Sci..

[111] Bejerano G, Lowe CB, Ahituv N, King B, Siepel A, Salama SR, Rubin EM, James Kent W, Haussler D (2006). A distal enhancer and an ultraconserved exon are derived from a novel retroposon. Nature..

[112] Nakanishi A, Kobayashi N, Suzuki-Hirano A, Nishihara H, Sasaki T, Hirakawa M (2012). A SINE-derived element constitutes a unique modular enhancer for mammalian diencephalic Fgf8. Plos One..

[113] Nishihara H, Kobayashi N, Kimura-Yoshida C, Yan K, Bormuth O, Ding Q, Nakanishi A, Sasaki T, Hirakawa M, Sumiyama K, Furuta Y, Tarabykin V, Matsuo I, Okada N (2016). Coordinately co-opted multiple transposable elements constitute an enhancer for wnt5a expression in the mammalian secondary palate. Plos Genet..

[114] Nishihara H, Smit AFA, Okada N (2006). Functional noncoding sequences derived from SINEs in the mammalian genome. Genome Res..

[115] Samstein RM, Josefowicz SZ, Arvey A, Treuting PM, Rudensky AY (2012). Extrathymic generation of regulatory T cells in placental mammals mitigates maternal-fetal conflict. Cell..

[116] Lam DD, de Souza FSJ, Nasif S, Yamashita M, López-Leal R, Otero-Corchon V (2015). Partially redundant enhancers cooperatively maintain mammalian Pomc expression above a critical functional threshold. Plos Genet..

[117] Martin M (2011). Cutadapt removes adapter sequences from high-throughput sequencing reads. EMBnet.journal..

[118] Langmead B, Salzberg SL (2012). Fast gapped-read alignment with Bowtie 2. Nat Methods..

[119] Langmead B, Trapnell C, Pop M, Salzberg SL (2009). Ultrafast and memory-efficient alignment of short DNA sequences to the human genome. Genome Biol..

[120] Li H, Handsaker B, Wysoker A, Fennell T, Ruan J, Homer N (2009). The Sequence Alignment/Map format and SAMtools. Bioinformatics..

[121] Feng J, Liu T, Qin B, Zhang Y, Liu XS (2012). Identifying ChIP-seq enrichment using MACS. Nat Protoc..

[122] Quinlan AR, Hall IM (2010). BEDTools: a flexible suite of utilities for comparing genomic features. Bioinformatics..

[123] Ramírez F, Dündar F, Diehl S, Grüning BA, Manke T (2014). deepTools: a flexible platform for exploring deep-sequencing data. Nucleic Acids Res..

[124] Sievers F, Wilm A, Dineen D, Gibson TJ, Karplus K, Li W (2011). Fast, scalable generation of high-quality protein multiple sequence alignments using Clustal Omega. Mol Syst Biol..

[125] Waterhouse AM, Procter JB, Martin DMA, Clamp M, Barton GJ (2009). Jalview Version 2-a multiple sequence alignment editor and analysis workbench. Bioinformatics..

[126] Bailey TL (2011). DREME: motif discovery in transcription factor ChIP-seq data. Bioinformatics..

[127] Grant CE, Bailey TL, Noble WS (2011). FIMO: scanning for occurrences of a given motif. Bioinformatics..

[128] Price MN, Dehal PS, Arkin AP (2010). FastTree 2 - approximately maximum-likelihood trees for large alignments. Plos One..

[129] Yu G, Lam TT-Y, Zhu H, Guan Y (2018). Two methods for mapping and visualizing associated data on phylogeny using Ggtree. Mol Biol Evol..

