# Supplementary material for: Evolution of mouse circadian enhancers from transposable elements
Source: Genome Biol. 2021 Jun 29;22:193. doi: 10.1186/s13059-021-02409-9 (PMC8240256; doi:10.1186/s13059-021-02409-9)
Supplement: Supplementary file 1 — Additional file 1. Contains Supplementary Figures S1–S6. [file 13059_2021_2409_MOESM1_ESM.pdf]

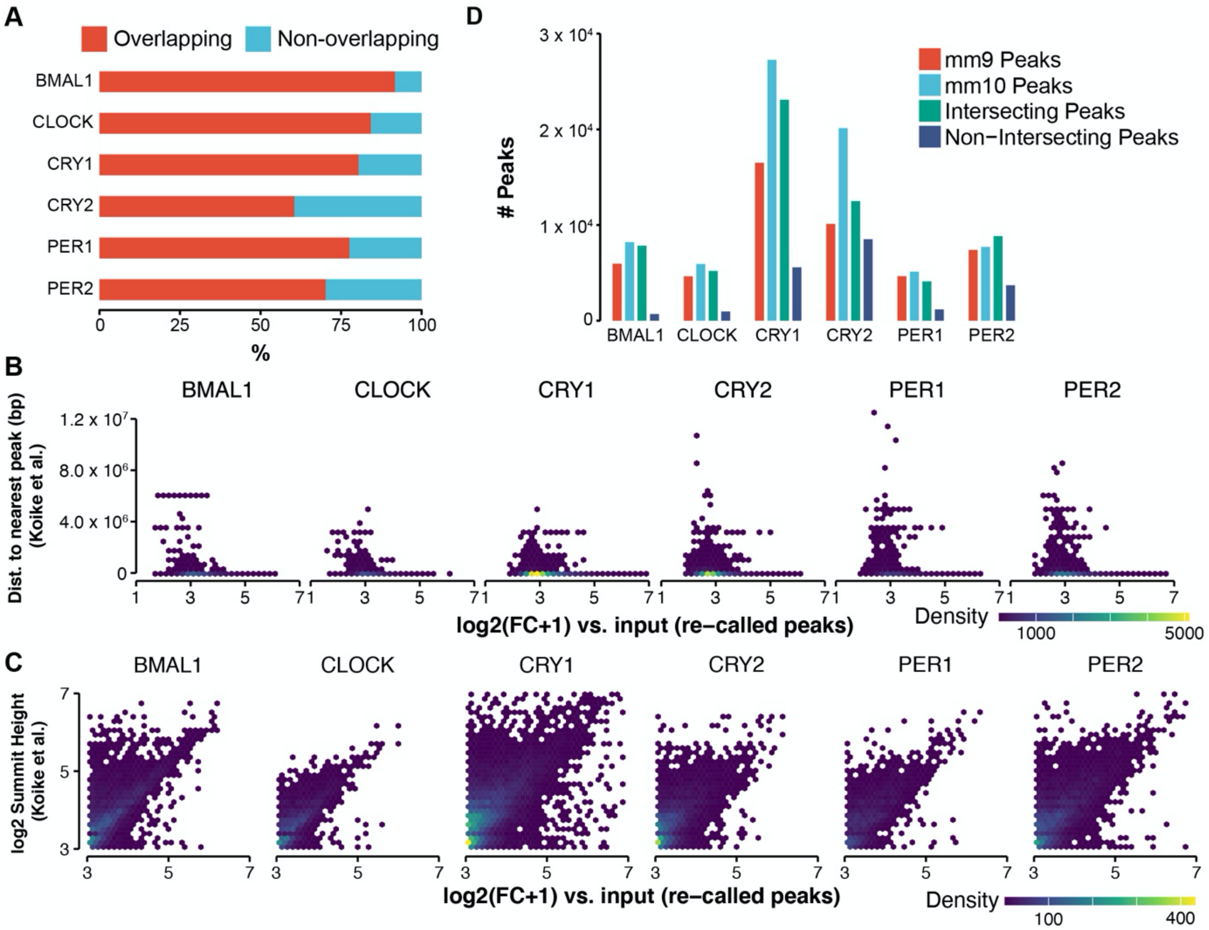

Fig. S1: New analysis of Circadian Regulator ChIP-seq data is similar to published results.

- **(A)** Percentage of peaks determined in the new analysis described in this report that overlap with peaks in the published set.
- **(B)** Fold-change as above for each peak in the new analysis compared to the distance to the nearest peak in the published set (peaks in the published set were mapped to mm10 coordinates using LiftOver).
- **(C)** Fold-change as reported by macs2 in the re-analysis compared to summit height for each peak as reported in the published set of peaks.
- **(D)** Number of peaks in the published set and in the new analysis presented here for each CR, as well as the number of peaks which are shared or not shared between the two datasets.

For **(C–D)**, the plot space was divided into hexbins and color-scaled according to the density of points in each bin to accommodate over-plotting.

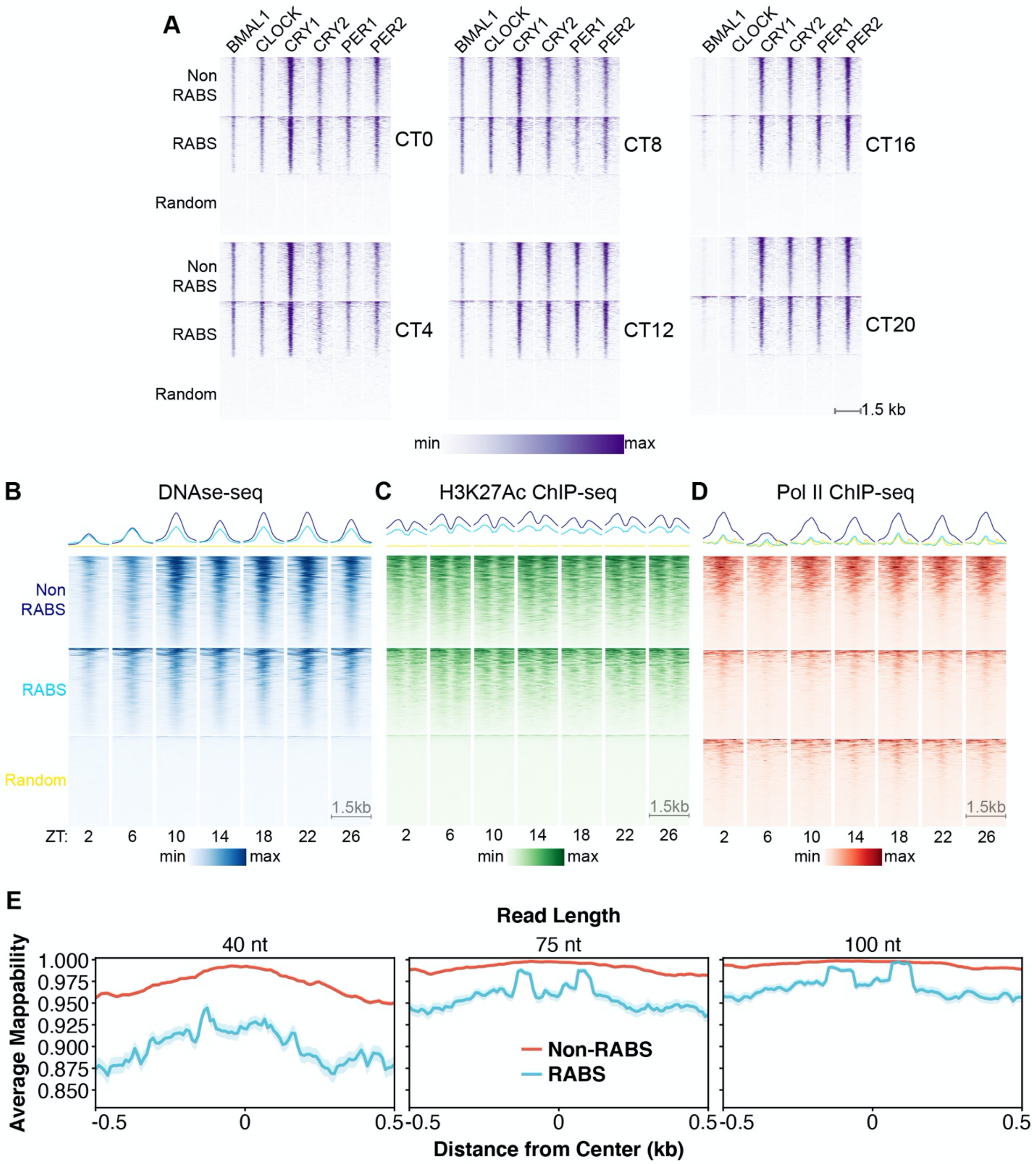

Fig. S2: RABS have a similar pattern of CR occupancy, but different chromatin properties compared to Non-RABS.

- **(A)** ChIP-seq signal of each CR centered at BMAL1 ChIP-seq peaks at RABS (n=1014), randomly selected Non-RABS (n=1014), and randomly selected repeats matching the familial composition of RABS (n=1014) over Circadian Time (CT).
- **(B)** DNase-seq signal over Zeitgeber time (ZT). Regions as in **(A)**.
- **(C)** H3K27Ac ChIP-seq over ZT. Regions as in **(A)**.
- **(D)** Pol II ChIP-seq at over ZT. Regions as in **(A)**.
- **(E)** Average mappability of BMAL1 Non-RABS and RABS at different read lengths.

In all panels, heatmaps are sorted by mean signal intensity across all rows in a block of heatmaps. Color scaling is min-max within each block.

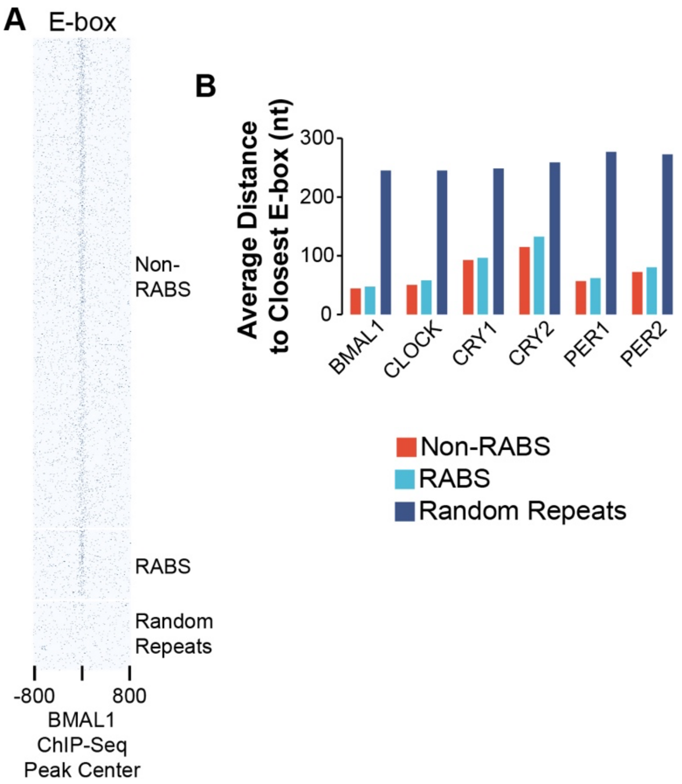

Fig. S3: Repeats contribute E-Box motifs.

- **(A)** Occurrence of E-Box motifs at RABS and Non-RABS BMAL1 ChIP-seq peaks, as well as a set of repeats randomly selected to match the familial composition of repeats associated with BMAL1 RABS. Signal strength (color intensity) is relative to motif similarity to the consensus motif.
- **(B)** Average distance to the closest E-Box motif from RABS or Non-RABS ChIP-seq peak centers as well as a randomly selected set of repeats that matches the familial composition of each set of CR RABS.

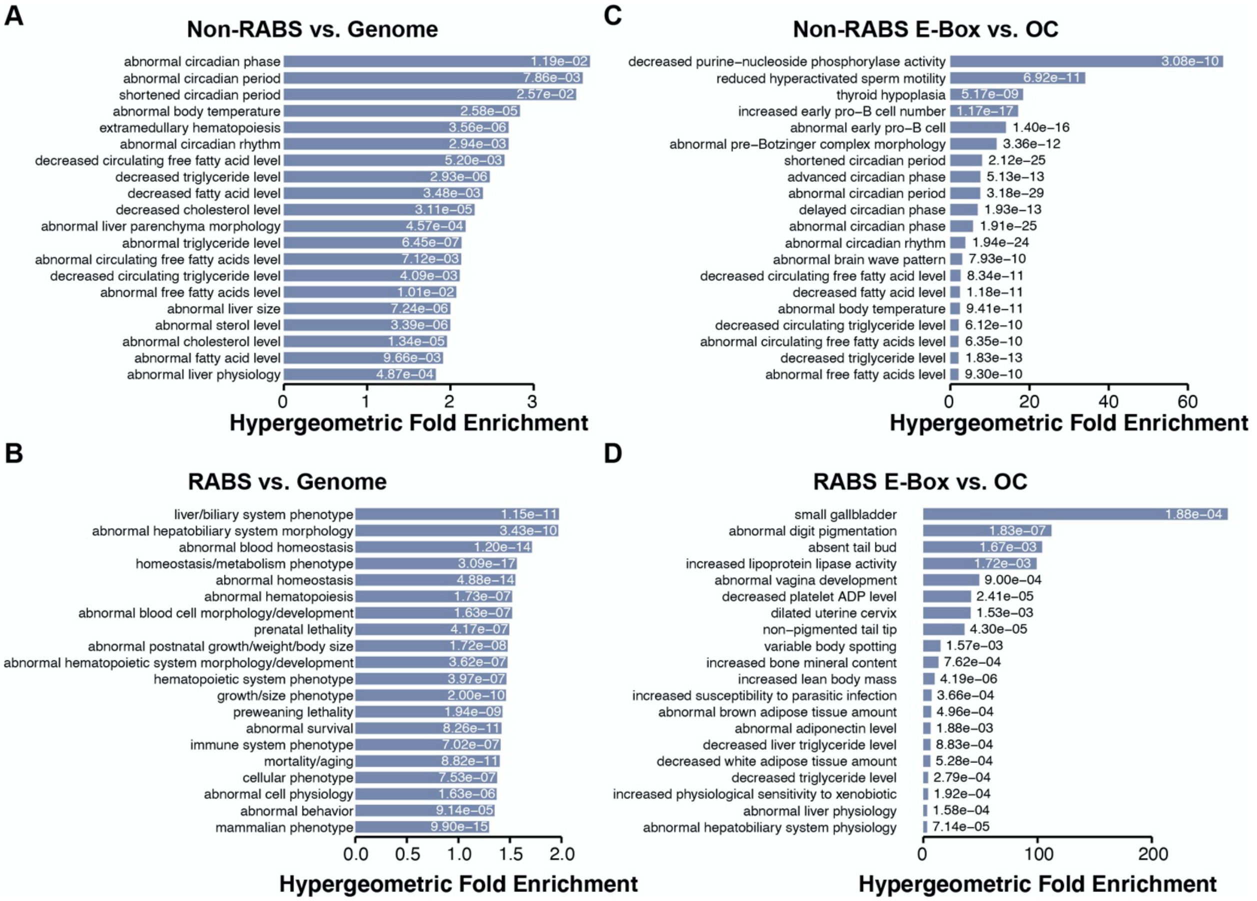

Fig. S4: RABS-proximal genes are enriched for more lineage-specific phenotypic associations than Non-RABS-proximal genes.

- **(A)** Phenotypic association enrichment of Non-RABS E-Box motifs using the entire mouse genome as background.
- **(B)** Phenotypic association enrichment of RABS E-Box motifs using the entire mouse genome as background.
- **(C)** Phenotypic association enrichment of Non-RABS E-Box motifs using open chromatin regions defined by DNase accessibility at any timepoint as background
- **(D)** Phenotypic association enrichment of RABS E-Box motifs using open chromatin regions defined by DNase accessibility at any timepoint as background

Enrichments were calculated by the tool GREAT using the two nearest genes within 1 Mb of each E-Box motif. FDR corrected hypergeometric q-values are either overlaid or adjacent to each bar.

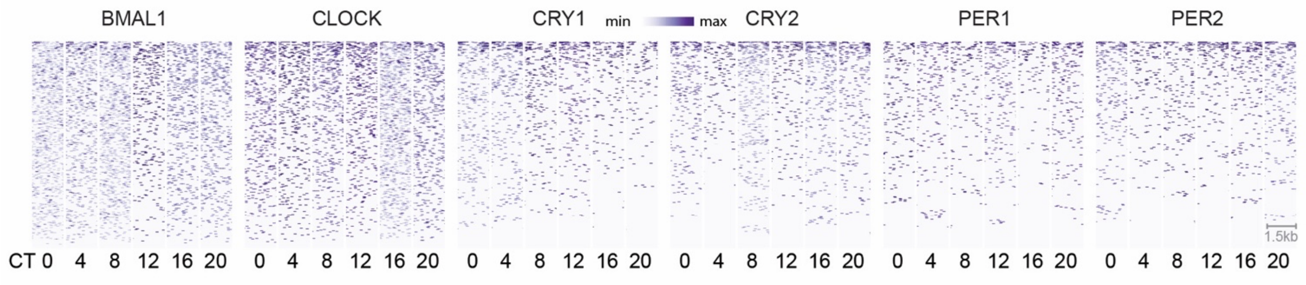

Fig. S5: RSINE1 elements not in open chromatin do not display oscillatory CR ChIP-seq signal.
ChIP-seq signal for each CR across circadian time (CT) at a randomly selected set of 328 RSINE1 elements which are not bound by CRs and do not reside in open chromatin regions as defined by DNase-seq. Heatmaps are centered at the middle of each element and extend 750 bp in each direction, and are sorted by mean signal intensity across all rows for a given CR. Color scaling is relative to min-max signal within each block of heatmaps.

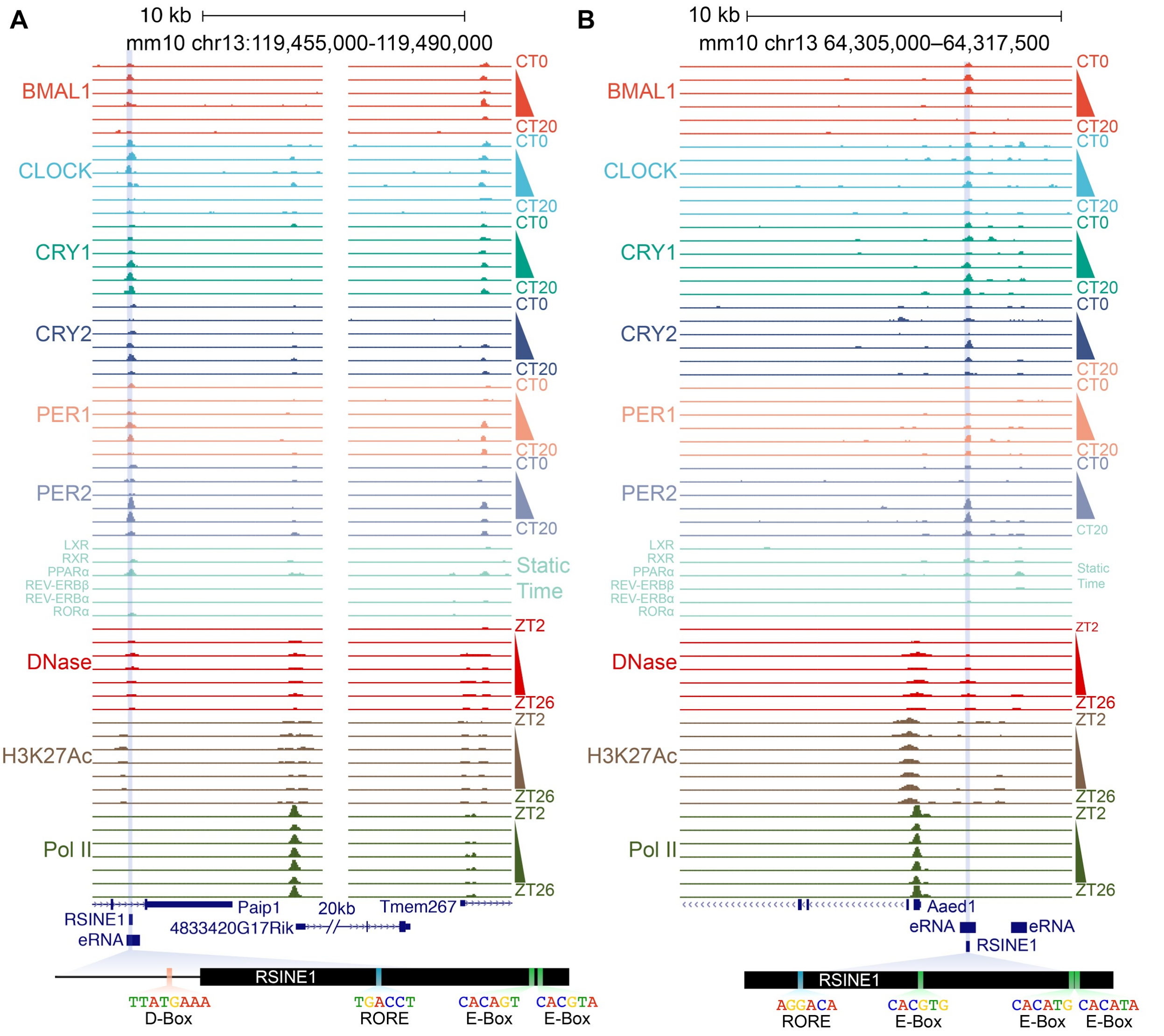

Fig. S6: UCSC genome browser screenshots of the two RSINE1s selected for luciferase reporter assays.

(A) Browser shot of the *Tmem267* locus.

(B) Browser shot of the *Aaed1* locus.

In each panel, coordinates of oscillating eRNAs are shown alongside the location of an overlapping CR-bound RSINE1. The location of the RSINE1 cloned into the luciferase reporter in Fig. 6 is highlighted, and the motif content of that particular element is shown. The *Tmem267* RSINE1 shows a small upstream region where a D-Box motif was found adjacent to the insertion site.
