# Supplementary material for: Evolution of mouse circadian enhancers from transposable elements
Source: Genome Biol. 2021 Jun 29;22:193. doi: 10.1186/s13059-021-02409-9 (PMC8240256; doi:10.1186/s13059-021-02409-9)
Supplement: Supplementary file 2 — Additional file 2. Review history. [file 13059_2021_2409_MOESM2_ESM.docx]

**Review history**

**First round of review**

**Reviewer 1**

The manuscript by Judd, Sanderson and Feschotte, entitled "Evolution of mouse circadian enhancers from transposable elements", presents compelling data on the origins of TE enhancers. The authors use published NGS data pertaining to circadian gene regulatory network to demonstrate that a specific family of TEs, RSINE1, is pre-wired to contain binding sites to reinforce and potentially rewire such network. The manuscript is well written and highly pedagogic for the non-TE community so I would like to congratulate the authors for such effort. Every time I wanted to see an experiment (mappability issues, consensus sequences binding sites, orthologous regions…) the authors had done it. One thing that I find important is the idea that enhancers derived from TEs are potentially redundant with non-TE enhancers, or weak, and their depletion might not cause major changes in regulatory networks. I think this is important since, in my opinion, TEs have gone from dark matter to gold matter, and it is important to see things in the light of evolution, and therefore, of selection, as the authors do it. Below are a few specific comments on the manuscript:

1)     The legend for figure S1B & C are inverted
2)     Figure 1C is confusing when compared to the text. The significance showed above the graphs are RABS vc non-RABS I suppose, but this should be added clearly (readers could mistake it to  timepoint differences). It is not easy to read increase and decrease of the ChIP signal a part from obvious cases (BMAL1 16-20 decrease). Could the authors show number of BSs instead/or with ChIP-seq signal so it is more obvious?
3)     Regarding mappability and RABS binding sites being smaller, I think a mappability plot would clear this better than just suggesting the read sizes are different. Also the authors could comment on the DNA fractionation methods used for the different assays because this might be an issue when mapping reads to euchromatin/heterochromatin.
4)     When the authors impose >50 bound copies, it would be interesting, for the readers and non-TE people, to have an idea of how many copies within a family that is. The RSINE1, if I am not mistaken, has thousands of copies, so it really puts things into perspective to see how little are indeed bound by the circadian TF (even though such bindings are indeed enriched).
5)     Figure S5 is missing a figure legend in the graph
6)     I have issues with the concept of ancestral vs consensus sequence. Indeed, the consensus sequence illustrates the average sequence in a pool of TE copies, but if different burst have occurred successfully then the consensus sequence might simply show the average sequence for the most recent, and massive burst. I would also use consensus sequences as you did, but adding a sentence to the paper might clarify such notions for the readers. Also, could the authors do a K2P landscape plot for the RSINE1s? This would emphasize the idea that RSINE1 have indeed colonized the mouse genome in a rapid burst of transposition.
7)     Regarding the orthologous regions you searched in rat, any convergent evolution observed in other TEs? Or even other regions? What I mean is : on the orthologous regions in rat that are devoid of RSINE1, or the RSINE1 has no BS, are there other additional BSs nearby?
8)     Figure 5A: what is the distance depicted in the schemes?
9)     Finally, I don't get the model where "most insertions were neutral or mildly deleterious". I guess we don't know what "most insertions were" but we know that the retained ones are neutral or mildly deleterious...
10)     And, my last comment, is curiosity. Have the authors tried to look at some of their RSINE1 BS in other mice strains? Specially if they are associated with pigmentation, as we know the different mice strains can have different coat color, tail color etc?

**Reviewer 2**

Judd and coworkers mined published data sets (genome sequences; ChIP-seq, RNA-seq, Dnase1-seq, etc) to examine the possibility that the invasion of repetitive elements into transcriptional regulatory regions participate in the control of circadian gene expression in liver. Their analysis revealed that RSINE1 elements, which are short interspersed retrotransposons, were enriched for binding sites occupied by circadian regulators. The sequences of RSINE1 elements already contain sequences with similarities to E-boxes and orphan nuclear receptor response elements, both known to play important roles in driving circadian transcription. The authors propose that in some cases the degenerate "pre-motifs" evolved into binding sites with higher affinities. C to T transitions, driven by the deamination of 5-methylated cytosines in CpG dinucleotides, might have contributed to this process, as RSINE1 elements are highly methylated in inactive regions of the genome. C/T transitions are somewhat enriched in E.box binding sites for BMAL1-CLOCK complexes, which are the positive limb members within the primary negative feedback loop. These complexes generate daily oscillations in the transcription of the negative limb members (Cry's and Per's). The authors conclude, however, that repeat-associated binding sites (RABS) play a less important role than non-repeat-associated binding sites (non-RABS) in the orchestration of circadian gene expression. In keeping with this conjecture, RABS are less well conserved than non-RABS during mammalian evolution.

Comments

(1) In the discussion section (lines 544-546) the authors write: Further study is needed to evaluate whether CR-bound TEs have directly contributed to changes in circadian gene expression with possible phenotypic consequences. Thereby they hint to the major problem of their paper, namely that there is little, if any, evidence that the short retrotransposons really contribute functionally to circadian gene transcription. In Figure 6A, the authors display some results obtained in transfection experiments with luciferase reporter genes. These assays, which are the only experiments in the paper testing the functional significance of RSINE1 elements, suggest that RSINE1 may affect the magnitude of reporter gene expression. However, in contrast to the cartoon shown in Fig. 6B, the transiently transfected plasmids do not assume a bona fide chromatin structure resembling that of genes. Moreover, these transfection experiments do not address the central question of whether RSINE1 elements participate in the control of cyclic transcription.

(2) On page 29 (lines 444-446) the authors refer to Fig. 6C and state: "This revealed that most elements which have developed perfect E-Box motifs (CACGTG) in the mouse have not developed the same motif in the rat (Fig. 6C)". (i) In the copy I downloaded there is no panel C in Fig. 6, and "Fig. 6C" should probably read "Fig. 5D". (ii) If indeed perfect E-boxes in the mouse genome are mostly absent in the orthologous rat elements and if the perfect E-boxes in the mouse genome were functionally relevant, the circadian expression of the genes carrying them should differ between these two species with regard to relative amplitude (max-min/max+min), and/or phase, and/or period length. In the human genome, the RABS binding sites are virtually absent, and the difference of circadian gene expression should be even more dramatic between rodents and humans. If none of these circadian parameters were different in cells of mice, rats and humans, the invasion of RSINE1's into murine enhancer/promoter regions of circadian genes would be of limited interest at best. In general, the addition of transcription factor binding sites to enhancers would be expected to elicit strong effects on transcription. Indeed, sequence-specific TFs, when bound to their functional cognate elements, frequently act in a synergistic fashion. Keep in mind that the free binding energy is logarithmically related to the binding affinity (ΔG0 = RTlnKD). Thus, at 37°C (310 °K) a gain (ΔΔG0) of only -1.4 kCal/mol in binding energy results in a 10x increase in affinity (i.e. 1/10 KD).

(3) The authors refer to Figure 7A (line 493) and Fig 8 (line 510). None of these Figures exist in the copy I downloaded. No reference is made to Fig. 5D in the text, as the authors confused Fig. 5D with Fig 6B (see above).

**Authors’ response to reviewers**

We thank the reviewers for their constructive comments that have helped us to improve this manuscript.

Reviewer #1: The manuscript by Judd, Sanderson and Feschotte, entitled "Evolution of mouse circadian enhancers from transposable elements", presents compelling data on the origins of TE enhancers. The authors use published NGS data pertaining to circadian gene regulatory network to demonstrate that a specific family of TEs, RSINE1, is pre-wired to contain binding sites to reinforce and potentially rewire such network.

The manuscript is well written and highly pedagogic for the non-TE community so I would like to congratulate the authors for such effort. Every time I wanted to see an experiment (mappability issues, consensus sequences binding sites, orthologous regions…) the authors had done it. One thing that I find important is the idea that enhancers derived from TEs are potentially redundant with non-TE enhancers, or weak, and their depletion might not cause major changes in regulatory networks. I think this is important since, in my opinion, TEs have gone from dark matter to gold matter, and it is important to see things in the light of evolution, and therefore, of selection, as the authors do it. Below are a few specific comments on the manuscript:

We thank the reviewer for their complimentary feedback and for recognizing the importance of our work! We have addressed their specific comments below:

1) The legend for figure S1B & C are inverted

Thank you for reading carefully, we have now swapped the legends to the correct order.

2) Figure 1C is confusing when compared to the text. The significance showed above the graphs are RABS vc non-RABS I suppose, but this should be added clearly (readers could mistake it to timepoint differences). It is not easy to read increase and decrease of the ChIP signal apart from obvious cases (BMAL1 16-20 decrease). Could the authors show number of BSs instead/or with ChIP-seq signal so it is more obvious?

We apologize for the confusion and acknowledge that the figure legends/text for this figure should have been clearer. The reviewer’s assumption that the significance test is RABS vs Non-RABS at each timepoint is correct, we have now clarified this by including “Kruskal-Wallis; RABS vs. Non-RABS” in the figure alongside the significance threshold key as well as in the legend for this panel (Fig 1; Fig 1C-D legend).

Unfortunately displaying number of binding sites doesn’t show much, because we are focused only on BMAL1 peaks for this analysis to have a consistent set of peaks for comparison across different types of data (the number of binding sites contributing to each red [Non-RABS; 7197] and blue [RABS; 1014] boxplot is the same across all timepoints and datasets for both figure 1C and 1D). Regarding ChIP-seq signal, we do actually show this as heatmaps in Fig S2. While we feel that these heatmaps of ChIP- seq signal are more illustrative, in the end we settled on using boxplots as the main text figure as we felt they were more quantitative. However, it was likely non-intuitive for the reader to make this connection, so we have now explicitly stated the connection between these two figures in the legend (Fig 1C-D legends)

We have also attempted to make both the text and the legend clearer regarding what this figure is and what the reader should be comparing. (line 163 & 217, Fig 1C-D legends).

We hope that these clarifications clear up any misunderstanding that the reviewer or any other reader may have had regarding this figure.

3) Regarding mappability and RABS binding sites being smaller, I think a mappability plot would clear this better than just suggesting the read sizes are different. Also the authors could comment on the DNA fractionation methods used for the different assays because this might be an issue when mapping reads to euchromatin/heterochromatin.

We have now included mappability plots for RABS and Non-RABS (see figure S2E and line 221). It is difficult to simulate mappability of paired-end reads, however, we demonstrate the general trend that mappability increases with read length at these sites.

Koike et al (CR TF ChIP-seq) stated that they fragmented chromatin by sonication 10 times of 30 seconds each. Sobel et al (H3K27Ac and Pol II ChIP-seq) stated that they fragmented chromatin by sonication 10 times of 15 seconds each. While it is true that different fractionation methods could cause differences in recovery of heterochromatic fragments (which don’t fragment as efficiently), we think that the methods used in both cases are likely not different enough to cause the differences we see in the intensity of the data at RABS vs Non-RABS. We prefer not to speculate on this in the manuscript, because there is also variance in fragmentation observed between different machines, parameters, etc., and without all this information (model of sonicator, 1.5 ml vs 15 ml tubes, intensity setting, polypropylene vs polystyrene tubes, etc.) it is very difficult to draw meaningful conclusions.

4) When the authors impose >50 bound copies, it would be interesting, for the readers and non-TE people, to have an idea of how many copies within a family that is. The RSINE1, if I am not mistaken, has thousands of copies, so it really puts things into perspective to see how little are indeed bound by the circadian TF (even though such bindings are indeed enriched).

There is a total of 115,806 RSINE1 insertions in the mouse genome. We agree that it is striking that even with only 328 bound elements, we still detect significant enrichment. We have now included this number in the text at line 254, indeed it puts things into perspective

5) Figure S5 is missing a figure legend in the graph

We have inspected Figure S5 carefully and do not see an omitted figure legend. We would be happy to update the figure with any missing information if presented with more information on the missing legend.

6) I have issues with the concept of ancestral vs consensus sequence. Indeed, the consensus sequence illustrates the average sequence in a pool of TE copies, but if different burst have occurred successfully then the consensus sequence might simply show the average sequence for the most recent, and massive burst. I would also use consensus sequences as you did, but adding a sentence to the paper might clarify such notions for the readers. Also, could the authors do a K2P landscape plot for the RSINE1s? This would emphasize the idea that RSINE1 have indeed colonized the mouse genome in a rapid burst of transposition.

Excellent points! We have now added the requested text and plot (lines 401-404; Fig 4I). The plot, which shows a normal distribution, further supports the notion that the RSINE1 family has amplified through a single, massive burst of transposition.

7) Regarding the orthologous regions you searched in rat, any convergent evolution observed in other TEs? Or even other regions? What I mean is : on the orthologous regions in rat that are devoid of RSINE1, or the RSINE1 has no BS, are there other additional BSs nearby?

This is an interesting question, however, there is not any publicly available BMAL1/CLOCK ChIP-seq data in the rat. Thus, it is difficult to look for nearby binding sites that have arisen by convergent evolution, as we don’t know for sure where any true binding sites are in the rat. We did check within a 4-kb window around the orthologous (bound in mouse) RSINE1 locations in the rat and detected no significantly enriched motifs. Since this is a negative result that is hard to interpret, we don’t wish to include this observation in the manuscript.

8) Figure 5A: what is the distance depicted in the schemes?

The distance depicted in the schemes is the median distance for each class of element. We have now noted this in the legend.

9) Finally, I don't get the model where "most insertions were neutral or mildly deleterious". I guess we don't know what "most insertions were" but we know that the retained ones are neutral or mildly deleterious...

We agree and did not intend to make a claim about the nature of non-retained insertions. Thus, we have revised this statement to “most fixed insertions were neutral or mildly deleterious…” (line 665)

10) And, my last comment, is curiosity. Have the authors tried to look at some of their RSINE1 BS in other mice strains? Specially if they are associated with pigmentation, as we know the different mice strains can have different coat color, tail color etc?

We were also curious about this! We’ve looked at some of the RSINE1 CR BS that overlap with oscillating eRNAs, and we do see some that contain SNPs between mouse strains. Here is one example:

In this example, the RSINE1 element contains 3 SNPs, and all occur within proto-motif regions. None of the motifs are perfect binding sites. The RORE has 3 suboptimal bases in the reference state, but there is a SNP in CAST, PWK, and SPRET strains that reduces this to 2 suboptimal bases. The first E-Box has one suboptimal base in the reference state, but the SPRET strain has two as a result of a SNP. Finally, the third E- Box has one suboptimal base in the reference state (and in the rat), which has changed to a perfect motif in the CAST strain as the result of a T to C mutation. This element is located downstream of the *Tnk2* gene, and ~50kb upstream of the *Muc3* promoter.

While these are potentially intriguing observations, they remain anecdotal, and it is difficult to predict how much these motif changes would impact CR binding and the regulatory function of such element. We would need functional genomics data across multiple strains to assess the impact of these variants. We feel that at this point these observations are too preliminary to include in the manuscript, and further detailed analysis falls beyond the scope of this paper.

Reviewer #2: Judd and coworkers mined published data sets (genome sequences; ChIP-seq, RNA-seq, Dnase1-seq, etc) to examine the possibility that the invasion of repetitive elements into transcriptional regulatory regions participate in the control of circadian gene expression in liver. Their analysis revealed that RSINE1 elements, which are short interspersed retrotransposons, were enriched for binding sites occupied by circadian regulators. The sequences of RSINE1 elements already contain sequences with similarities to E-boxes and orphan nuclear receptor response elements, both known to play important roles in driving circadian transcription. The authors propose that in some cases the degenerate "pre-motifs" evolved into binding sites with higher affinities. C to T transitions, driven by the deamination of 5-methylated cytosines in CpG dinucleotides, might have contributed to this process, as RSINE1 elements are highly methylated in inactive regions of the genome. C/T

transitions are somewhat enriched in E.box binding sites for BMAL1-CLOCK complexes, which are the positive limb members within the primary negative feedback loop. These complexes generate daily oscillations in the transcription of the negative limb members (Cry's and Per's). The authors conclude, however, that repeat-associated binding sites (RABS) play a less important role than non-repeat-associated binding sites (non-RABS) in the orchestration of circadian gene expression. In keeping with this conjecture, RABS are less well conserved than non-RABS during mammalian

evolution.

Comments

(1) In the discussion section (lines 544-546) the authors write: Further study is needed to evaluate whether CR-bound TEs have directly contributed to changes in circadian gene expression with possible phenotypic consequences. Thereby they hint to the major problem of their paper, namely that there is little, if any, evidence that the short retrotransposons really contribute functionally to circadian gene transcription. In Figure 6A, the authors display some results obtained in transfection experiments with luciferase reporter genes. These assays, which are the only experiments in the paper testing the functional significance of RSINE1 elements, suggest that RSINE1 may affect the magnitude of reporter gene expression. However, in contrast to the cartoon shown in Fig. 6B, the transiently transfected plasmids do not assume a bona fide chromatin structure resembling that of genes. Moreover, these transfection experiments do not address the central question of whether RSINE1

elements participate in the control of cyclic transcription.

We agree with the reviewer that we present little functional evidence that RSINE1 is actually contributing to circadian gene transcription. However, the goal of our study is not to provide evidence that RSINE1 elements are controlling mouse circadian gene expression nor that they have altered circadian gene expression during evolution.

Nowhere in the paper do we make such claims. Rather, the study shows that these elements display all the biochemical features of circadian enhancers and we dissect in detail how these activities emerged during evolution. Regardless of the function (or lack thereof) of these activities, we believe our findings will be of interest to scientists interested in cis-regulatory evolution, as reviewer #1 has noted.

Having said that, we do present some data that hints that at least some RSINE1 elements may be in fact driving cyclic transcription. For instance, we identified CR- bound RSINE1 elements for which the nearest gene is known to be transcribed cyclically, and furthermore 37 of the CR-bound RSINE1s show oscillatory nascent transcription (by GRO-seq); a gold standard in detecting active transcriptional regulatory elements. Given these findings, we do not think it is unreasonable to speculate that a subset of CR-bound RSINE1s may actually be functionally contributing to circadian gene expression in the mouse liver. The elements we tested in reporter assays were selected precisely because they overlap with regions that have been shown to be rhythmically transcribed *in vivo.*

The reviewer’s statement that “transiently transfected plasmids do not assume a bona fide chromatin structure resembling that of genes” is debatable and there are conflicting reports in the literature about this [PMID: 3859838, 7510391]. Notwithstanding these controversies, the purpose of these assays in our study was to examine the regulatory potential of these elements, not to make conclusions about their function *in vivo*. We concede that there were certain phrasings in the text which may have given this reviewer the mistaken impression that we intended to prove that these RSINE1-derived circadian regulatory elements are functional and drive cyclic transcription. We have rephrased key sentences in the results and discussion to be more specific about our claims (lines 570-572, 668, 669, 516, 532, 535). For example:

Initial Submission (line 570)

Further study is needed to evaluate whether CR-bound TEs have directly contributed to changes in circadian gene expression.

Revision (line 570)

**However, we have not functionally demonstrated that RSINE1 elements drive cyclic transcription in vivo, and as a result** further study is needed to evaluate whether CR-bound TEs have directly contributed to changes in circadian gene expression.

Initial Submission (line 668)

…a small subset of elements were tolerated or advantageous due to their proximity to existing circadian regulatory elements and have been co-opted as circadian enhancers either through binding site turnover … or rarely by wiring a new gene into the circadian regulatory network (Fig 6B).

Revision (line 668)

…a small subset of elements were tolerated or advantageous due to their proximity to existing circadian regulatory elements and **may** have been co-opted as circadian enhancers either through binding site turnover … or rarely **could have wired** a new gene into the circadian regulatory network (Fig. 6B).

We do agree with the reviewer that functional experiments directly testing the role of these RSINE1 elements in circadian gene expression *in vivo* would be interesting.

However, this would be highly laborious as it would require making knockout mice, which we have neither the expertise nor approval to do. Furthermore, establishing the functional role of these elements is beyond the scope of this study, which again we clearly framed in Introduction. We think our paper represents a significant advance in the field regardless of the extent by which these elements have impacted the evolution of circadian gene expression

(2) On page 29 (lines 444-446) the authors refer to Fig. 6C and state: "This revealed that most elements which have developed perfect E-Box motifs (CACGTG) in the mouse have not developed the same motif in the rat (Fig. 6C)". (i) In the copy I downloaded there is no panel C in Fig. 6, and "Fig. 6C" should probably read "Fig. 5D".

The reviewer is correct that we intended to refer to Fig 5D. We have corrected this typo.

(ii) If indeed perfect E-boxes in the mouse genome are mostly absent in the orthologous rat elements and if the perfect E-boxes in the mouse genome were functionally relevant, the circadian expression of the genes carrying them should differ between these two species with regard to relative amplitude (max-min/max+min), and/or phase, and/or period length. In the human genome, the RABS binding sites are virtually absent, and the difference of circadian gene expression should be even more dramatic between rodents and humans. If none of these circadian parameters were different in cells of mice, rats and humans, the invasion of RSINE1's into murine

enhancer/promoter regions of circadian genes would be of limited interest at best. In general, the addition of transcription factor binding sites to enhancers would be expected to elicit strong effects on transcription. Indeed, sequence-specific TFs, when bound to their functional cognate elements, frequently act in a synergistic fashion. Keep in mind that the free binding energy is logarithmically related to the binding affinity (ΔG0

= RTlnKD). Thus, at 37°C (310 °K) a gain (ΔΔG0) of only -1.4 kCal/mol in binding energy results in a 10x increase in affinity (i.e. 1/10 KD).

First, as discussed above in response to point (1), our manuscript does not attempt to examine the functional consequence of the cis-regulatory elements introduced by RSINE1. Second, the reviewer does not provide any actionable suggestions for improvement, and as a result we have not modified our manuscript in response to this comment.

We agree it would be interesting if what the reviewer proposes is true regarding cyclic expression in rat/human of genes nearby CR-bound RSINE1s in the mouse.

Unfortunately, to our knowledge, there is no publicly available circadian gene expression profiling data in the rat, and for reasons outlined above we feel it would be onerous and beyond the scope of this study for us to perform these experiments in rat for the purpose of this revision. These experiments would be impossible to perform in humans for obvious reasons. Thus, what the reviewer seems to be getting at here would require a whole new experimental study.

Furthermore, we do not agree with many of the reviewer’s assertions. In particular, we do not think that one should necessarily always expect that the addition of a single TFBS motif would “elicit strong effects on transcription”. The evidence in the literature is mixed, and suggests that the consequences of the addition/deletion of a single TFBS motif to a regulatory element can range from virtually inconsequential to dramatic, and likely depend on a number of factors including the affinity of the binding site, the number of redundant binding sites, and the degree of co-operativity with other TFs [PMID: 1870969, 15696541, 8548797, 25557079]. Regulatory elements include clusters of binding sites, some optimal and others sub-optimal. There is often built-in redundancy. In fact, it has been reported multiple times that the deletion of an entire enhancer element had little impact on the expression of its target gene [PMID: 26743005, 33482114, 20512118, 27863239, 11911829] likely because there is often redundancy among regulatory elements for the same gene just as there is often redundancy for individual TFBSs within a single enhancer.

Given our finding that RSINE1 elements preferentially developed circadian TF binding activity when nearby existing circadian TF binding sites, we would actually predict that most of these RSINE1-derived regulatory elements likely have limited to no functional consequence on their own, but rather act as redundant enhancers or have facilitated binding site turnover events or fine-tuning of existing enhancers. We disagree that this lack of straightforward function means these elements are of “limited interest”. We strongly believe many in both the transcription regulation field and transposable elements field who would consider our findings of great interest. Our results underscore the complexity of the mammalian cis-regulatory lexicon and provide an alternate paradigm for the co-option of transposable elements as host regulatory elements.

(3) The authors refer to Figure 7A (line 493) and Fig 8 (line 510). None of these Figures exist in the copy I downloaded. No reference is made to Fig. 5D in the text, as the authors confused Fig. 5D with Fig 6B (see above).

We apologize for the confusion; these were remnants from a previous draft that had a different figure layout. We have corrected the typos.

**Second round of review**

**Reviewer 1**

The authors have successfully addressed all my comments.

**Reviewer 2**

In their rebuttal letter the authors point out that their paper did not address the issue of whether the transposable element-derived putative cis-acting elements are functionally relevant. They even speculate that most of them are not, due to redundance. Hence, I cannot extract a crisp and well-supported take-home message and maintain my opinion that this bioinformatic analysis is of limited interest.
